# Crystallization of Polycaprolactone within Nanopapers Based on Graphene-Related Materials

**DOI:** 10.1021/acs.macromol.5c00752

**Published:** 2025-06-30

**Authors:** Hui Zhao, Ricardo A. Pérez-Camargo, Giacomo Damonte, Marco Armandi, Orietta Monticelli, Guoming Liu, Alejandro J. Müller, Alberto Fina

**Affiliations:** † Dipartimento di Scienza Applicata e Tecnologia, Politecnico di Torino, Alessandria campus, viale Teresa Michel, 5, Alessandria 15121, Italy; ‡ POLYMAT and Department of Polymers and Advanced Materials: Physics, Chemistry and Technology, Faculty of Chemistry, University of the Basque Country UPV/EHU, Paseo Manuel de Lardizabal 3, Donostia-San Sebastián 20018, Spain; § Dipartimento di Chimica e Chimica Industriale, 9302 Università di Genova, Via Dodecaneso 31 Genova 16146, Italy; ∥ Dipartimento di Scienza Applicata e Tecnologia, Politecnico di Torino, Corso Duca degli Abruzzi 24, Torino 10129, Italy; ⊥ Beijing National Laboratory for Molecular Sciences, Institute of Chemistry, 53030 Chinese Academy of Sciences, Beijing 100190, China; # University of Chinese Academy of Sciences, Beijing 100049, China; ∇ IKERBASQUE, Basque Foundation for Science, Plaza Euskadi 5, Bilbao 48009, Spain

## Abstract

The crystallization of polymers in nanopapers based on graphene-related materials (GRMs) influences both elastic deformability and heat transfer within the nanostructure. Polycaprolactone (PCL) crystallizes in nanopapers, producing different crystalline fractions. We relate their formation to the interactions between polymer chains and the surface of GRM. In addition to conventional PCL crystals, we observe higher melting point crystals that result from strong heterogeneous nucleation, as well as crystals that melt above PCL’s equilibrium melting temperature, seemingly linked to the prewetting of crystalline layers. The relative intensity of various melting peaks depends on the structural features and defects of GRM and the nanopaper preparation process. The molecular weight of PCL affects the thermal stability of crystals that melt above PCL’s equilibrium melting temperature. Notably, these high-stability crystals cannot be thermally fractionated by successive self-nucleation and annealing (SSA), nor can they be dissolved in a conventional solvent for PCL, indicating a particularly strong interaction between PCL and GRM in nanopapers, which might be utilized in other hybrid organic–inorganic nanostructures.

## Graphical abstract

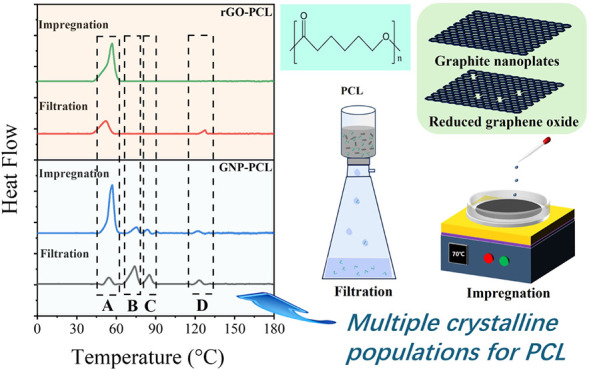

## 1 Introduction

Nanopapers are well-known structures consisting of nanofibers, nanoparticles, nanosheets, and other materials. These structures may exhibit excellent properties, such as lightweight,^1,2^ high strength,^3–5^ and high thermal conductivity.^6–9^ Graphene-related materials (GRMs) are known for their excellent thermal conductivity,^10–13^ as well as their electronic,^14,15^ corrosion-resistant,^16,17^ oxidation-resistant,^18,19^ and mechanical properties.^20–22^ GRMs include graphene oxide (GO), reduced graphene oxide (rGO), multilayer graphene (MLG), and graphene nanoplates (GNPs). GRMs have been used to prepare nanopapers, typically via suspension in a liquid, followed by vacuum-assisted filtration,^23–25^ and are mainly proposed for heat spreader applications.^26,27^ However, the inherent brittleness of nanopapers made from pristine GRM somewhat limits their effectiveness as heat spreaders. For this reason, the use of polymer binders was previously proposed.^28–30^ Polymers can act as effective adhesives between graphene nanosheets. However, common polymers exhibit extremely low thermal conductivity, typically ranging between 0.1 and 0.5 W/(mK),^31,32^ which is detrimental to the thermal conductivity of polymer composites. Generally, semicrystalline polymers have a higher thermal conductivity than amorphous polymers because the disordered chain conformation of amorphous polymers reduces the phonon mean free path and enhances the scattering of phonons,^33^ meaning that the higher the crystallinity, the greater the thermal conductivity.^33–35^

Polycaprolactone (PCL) is a semicrystalline biodegradable polymer. It is well-known for its room-temperature ductility and relatively high crystallinity, making it a good candidate for thermally conductive GRM-based nanocomposites.^36–39^ Furthermore, GRMs were reported to exhibit a strong nucleation effect, significantly increasing the crystallization temperature and crystallization rate of PCL.^39–42^ In a previous work,^28^ PCL was exploited to prepare flexible GNP nanopapers for heat spreaders. The results showed that PCL improved the mechanical behavior of GNP nanopapers while yielding thermal conductivity values of up to 170 W/(mK), which can effectively compete with conventional metal heat spreaders. Interestingly, besides the strong nucleation effect, GNPs were found to promote a peculiar crystallization of PCL, with multiple crystalline populations assigned to conventional unoriented PCL crystals, oriented PCL crystals, and two additional highly stable PCL crystalline populations. This phenomenon was explained by the possible formation of a prefrozen crystalline layer, based on previous studies on model surfaces.^43–46^ Tariq et al. presented in situ AFM measurements of PCL prefrozen on molybdenum disulfide substrates and compared the results with studies of prefrozen PCL on graphite and polyethylene substrates. The results showed that the *T*_m,max_ (the melting temperature of all crystals present) of prefrozen PCL on molybdenum disulfide is essentially the same as that on graphite.^43,44^ Nevertheless, the factors contributing to the emergence of highly stable PCL components remain unclear.

Therefore, to further elucidate the reasons for the highly thermally stable structures formed by PCL in GRM, a detailed structural comparison of nanopapers based on GNP and rGO was conducted in this study. In particular, the distinct crystallization behaviors of PCL on GNP and rGO were studied through differential scanning calorimetry (DSC) and wide-angle X-ray scattering (WAXS). Furthermore, the effects of polymer content, molecular weight, and different preparation methods on PCL crystallization in nanopapers were studied. Finally, the thermal conductivity of PCL/GRM nanopapers was investigated to assess correlations with the GRM structure and polymer crystallinity. This research provides valuable insights into the various morphologies of crystalline structures and thermally stable configurations of polymers on GRM, specifically on nanopapers, and represents a promising route for developing other polymer/GRM composites.

## 2 Experimental Section

### 2.1 Materials

Polycaprolactone (PCL) samples with different molecular weights were employed. High molecular weight (*M*_n_ = 50000 g/mol) PCL CAPA 6500 was purchased from Ingevity. Intermediate molecular weight (*M*_n_ = 10000 g/mol) PCL was purchased from Merck. Furthermore, low molecular weight PCL oligomers (*M*_n_ = 4000 g/mol and *M*_n_ = 2000 g/mol) were synthesized according to a procedure previously reported elsewhere.^47,48^ PCL with different molecular weights is referred to as M50, M10, M4, and M2, respectively. For the oligomers, the corresponding ^1^H NMR and FT-IR assignments of the two polymers are given hereunder:

^1^H NMR, δ (300 MHz, CDCl_3_, 30 mg/mL, ppm): 4.06 (−C**H**_**2**_–O–C=O PCL chain, t); 3.65 (−C**H**_**2**_–OH PCL chain terminal, t); 2.30 (−C**H**_**2**_–C=O PCL chain, t); 1.66 (−C**H**_**2**_– PCL chain, m); 1.40 (−C**H**_**2**_– PCL chain, m); 1.31 (−C**H**_**2**_– 1-dodecyl chain of 1-dodecanol initiator, m); 0.88 (−C**H**_**3**_ dodecyl chain terminal of 1-dodecanol initiator, t).

FT-IR (ATR mode, powdered sample, cm^–1^): 3400 (**O–H** stretching PCL chain terminal); 1728 (**C=O** stretching PCL backbone); 1296 (**C–O** and **C–C** stretching PCL backbone); 1241 and 1170 (unsymmetrical/symmetrical **C–O–C** stretching PCL backbone).

GRMs used in this work were supplied by AVANZARE (Navarrete, La Rioja, Spain), namely GNP and rGO flakes obtained by previously reported synthetic procedures. In brief, GNPs were prepared via rapid thermal expansion of overoxidized intercalated graphite,^49^ whereas rGO was prepared by oxidation of natural graphite, tip sonication in a water solution, and subsequent thermal reduction at 1060 °C in an argon atmosphere.^50^ The complete characterization of both products was previously reported elsewhere.^51^

Dimethylformamide (DMF, ≥ 99.8%, Merck or Carlo Erba Reagents) and toluene (Tol, 99.8%, Merck) were used as received.

### 2.2 Preparation Methods

#### 2.2.1 Preparation of Nanopapers via One-Step Filtration

The preparation process of GRM/PCL nanopapers was previously reported.^28^ In brief, different amounts of PCL pellets (50 mg or 250 mg) were dissolved in 150 mL of DMF at 60 °C for 1 h to obtain solutions with varying PCL concentrations. Subsequently, 50 mg of GNP or rGO was dispersed in the PCL solutions using sonication treatment in pulsed mode (5 s on and 5 s off) for 30 min with power set at 30% of the full output power (500 W). This was accomplished with an ultrasonication probe (Sonics Vibracell VCX-750, Sonics & Materials Inc.) equipped with a 13 mm diameter Ti-alloy tip. For comparison, selected nanopaper formulations were prepared after 2.5 h of sonication time in pulsed mode (5 s on and 5 s off). The suspension was transferred into a filtration system equipped with a polyamide-supported membrane (0.45 μm nominal pore size, diameter 47 mm, Whatman) and left for different filtration times (Scheme 1). The filtration time was set to 5, 17, and 72 h, respectively. While 5 h is sufficient to filter the suspension, the cake obtained on the filter still retains a significant amount of polymer solution, which progressively decreases over time. Therefore, allowing the cake to drain the excess solution for an extended period leads to a lower PCL content in the nanopapers. As a standard procedure, a long filtration time (72 h) was applied to remove excess PCL solution, yielding low PCL contents in the dry nanopapers. Unless otherwise specified, this procedure was applied to the different grades of PCL (M50, M10, M4, and M2), which have varying molecular weights. Furthermore, selected nanopapers were prepared by reducing the filtration time to yield higher PCL contents, as detailed in Table 1. After filtration, the sample was removed along with the filter and placed in an oven to remove the residual solvent. The drying process was carried out at 70 °C for 2 h; then, the temperature was increased to 120 °C, and the sample was dried for 1 h. After this phase, the dried nanopapers were carefully peeled off the filter and consolidated by applying a 6-ton load for 30 min at room temperature (RT). The details of the preparation of nanopapers are reported in Table S1. Selected nanopapers were extracted with toluene in a Soxhlet apparatus for 12 h to remove excess PCL and air-dried at room temperature for 48 h before further characterization. Such nanopapers have been denoted as SE for Soxhlet extraction.

**Scheme 1:**
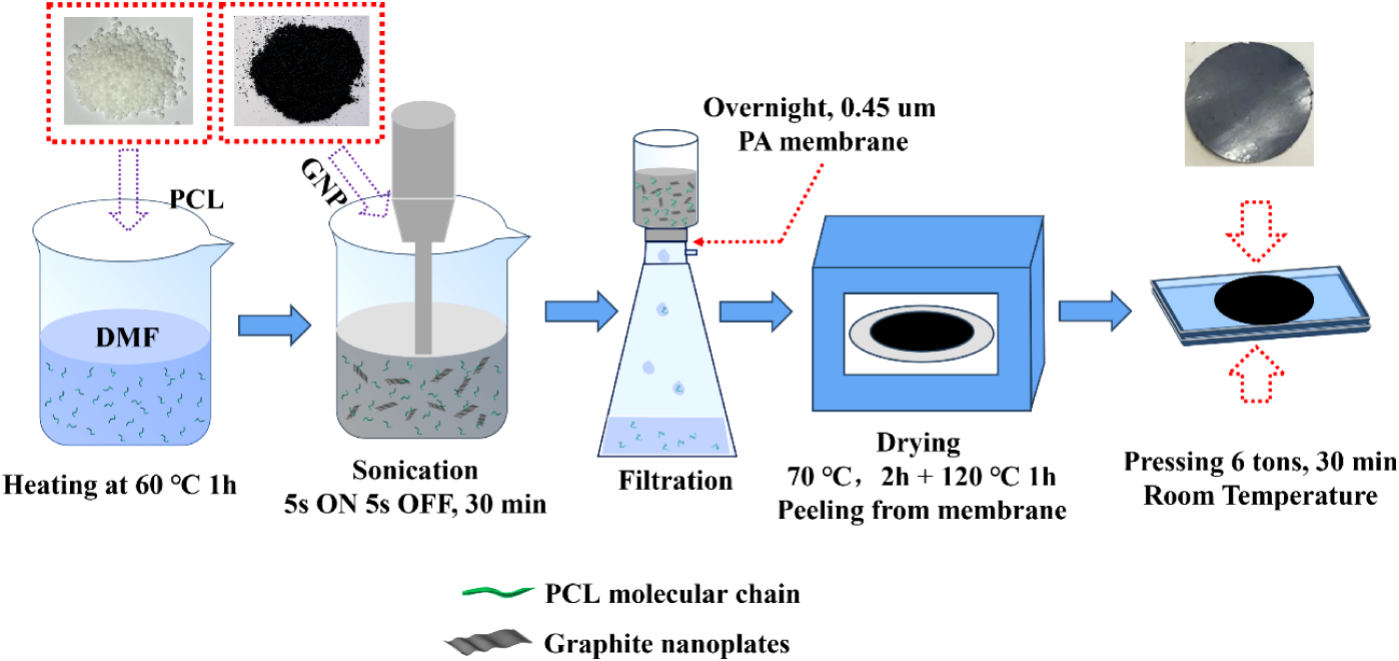
Filtration Process for Nanopaper Preparation

**Table 1:** PCL (M50) Content in Nanopapers and Thermal Stability from TGA Measurements, *DTG*_max_ Is the Temperature at Which the Sample Mass Loss Rate Is Maximum^a^

Formulation	Filtration time (h)	Weight percentage of PCL^b^ (wt %)	*DTG*_max_ (°C)
PCL	-	100	370
GNP:PCL 1:1	5	41.5 ± 3.4	406
	17	15.7 ± 2.8	401
	72	4.7 ± 1.0	387
GNP:PCL 1:5	5	79.4 ± 2.1	407
	17	27.7 ± 0.7	405
rGO:PCL 1:1	5	23.0 ± 3.5	409
	17	20.3 ± 0.9	403
	72	8.4 ± 1.0	392
rGO:PCL 1:5	5	61.6 ± 0.6	410
	17	51.5 ± 0.8	400

a The test samples were prepared by using the filtration method.

b Calculated as the mass loss observed after heating in nitrogen at 10 °C/min to 600 °C.

#### 2.2.2 Impregnation of GRM Nanopapers with PCL Solution

The preparation of GRM/PCL nanopapers via impregnation is done in two steps. First, pristine GRM nanopapers were prepared without PCL and without mechanical pressing, as shown in Scheme 1. In the second step, nanopapers were impregnated with a PCL solution and then mechanically consolidated. The impregnation process of GNP or rGO nanopapers is shown in Scheme 2. The desired PCL amount (typically about 10% of the nanopaper mass) was dissolved in 5 mL of DMF or toluene. The solution was then added dropwise to the surface of the GRM nanopaper, placed on a hot plate, and heated to 70 °C to remove the solvent slowly. Finally, the prepared samples were placed in an oven at 120 °C for 1 h to eliminate traces of the residual solvent. After this phase, the nanopapers were consolidated by compression under a load of 6 tons at RT for 30 min. The preparation details are reported in Table S2.

**Scheme 2:**
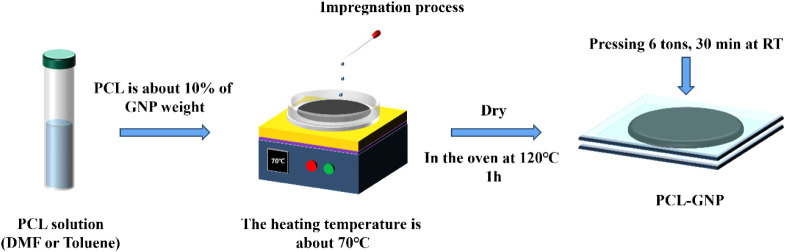
Impregnation Process of Nanopapers

### 2.3 Characterization Methods

Raman spectra were acquired using a Renishaw inVia Reflex (Renishaw PLC, UK) Raman microscope with an excitation laser wavelength of 514.5 nm, a power of 10 mW, and a resolution of 3 cm^–1^. For each GNP or rGO nanopaper, spectra were collected at three different points and averaged.

X-ray photoelectron spectroscopy (XPS) was performed on a VersaProbe 5000 Physical Electronics spectrometer with a monochromatic Al source and a hemispherical analyzer. Survey scans and high-resolution spectra were recorded with a spot size of 100 μm. Nanopapers were kept under vacuum overnight before the measurement to remove adsorbed molecules. A Shirley background function was utilized to adjust the spectra’s background. The C 1s peak fitting used the software CasaXPS and accounted for the contribution of C–C bonds with sp^2^-like characteristics using an asymmetric peak (Doniach-Šunjić shape),^52,53^ as previously calculated on freshly cleaved highly oriented pyrolytic graphite (HOPG) (ZYH grade, Mikromasch), with an obtained asymmetry index (α) of 0.115. Curve fitting employed a Gaussian (80%)-Lorentzian (20%) peak shape by minimizing the total square-error fit. The full width at half-maximum (fwhm) for each peak was maintained between 1.3 and 1.4 eV. The C 1s spectra were deconvoluted into several peaks: C–C sp^2^ with binding energy at 284.4 ± 0.1 eV, C–C sp^3^ at 285.0 ± 0.1 eV, C–OH at 285.7 ± 0.1 eV, C–O–C at 286.6 ± 0.2 eV, O–C=O at 288.0 ± 0.1 eV, C=O at 289.0 ± 0.1 eV, and the π-π* shakeup satellite peak from the sp^2^-hybridized C atoms at 291.0 ± 0.2 eV.^54–56^

Specific surface area and porous volume were measured using N_2_ adsorption/desorption isotherms at 77 K (ASAP2020 Plus - Micromeritics). The samples were previously outgassed at 373 K to remove water and other atmospheric contaminants. From the N_2_ isotherms, the specific surface area was measured by the Brunauer–Emmett–Teller (BET) multipoint method in the 0.10–0.25 relative pressure range; total porous volume (*V*_TOT_) was measured from the last adsorption isotherm point, and cumulative pore volume curves were obtained by applying the NLDFT method for carbon slit pores.

Scanning Electron Microscopy (SEM) micrographs were acquired by an EVO 15 SEM (Zeiss, Germany) with a beam voltage of 20 kV. The micrographs were obtained from the nanopaper’s cross-section. The nanopaper was placed in liquid nitrogen, allowed to cool thoroughly, and fractured by applying an external force.

Differential scanning calorimetry (DSC) tests were performed on a TA Q20 calorimeter (TA Instruments, USA). During the measurements, the samples were protected under a N_2_ atmosphere to avoid thermal oxidation. The heating/cooling rate was 10 °C/min. The reported enthalpy values were calculated based on the actual PCL content in PCL/GNP nanopapers, as determined by TGA analysis conducted after DSC testing of the samples. The crystallinity (*X*_C_) of PCL in different nanopapers was calculated by considering their real contents, ϕ_PCL_, following eq 1:

1 $$X_{C}\left(\%\right)=\frac{\Delta H_{m}}{\Delta H_{m}^{0}\times \Phi_{PCL}}\times 100$$

where Δ*H*_m_ is the measured heat of fusion, ϕ_PCL_ is the PCL content in the nanopapers, and $\Delta H_{m}^{0}$ is the melting enthalpy of 100% crystalline PCL (139.5 J/g).^57^

Successive self-nucleation and annealing (SSA) experiments were performed with DSC 8500 (PerkinElmer) connected to a liquid nitrogen cooling accessory (Intracooler 3). DSC was operated under a continuous ultrapure nitrogen flow of 20 mL/min to maintain an inert atmosphere. Calibration was performed using indium and zinc standards, employing a scanning rate of 20 °C/min and a sample mass of around 3 mg. The SSA experiments were conducted following the method established and reviewed by Müller et al.,^58–60^ with fractionation windows of 2.5 °C (for the highest temperature endothermic peaks) and 5 °C (for intermediate and low-temperature melting peaks) and holding times of 5 min at each temperature. This testing procedure and methodology for fractionating these samples have been previously elucidated in our earlier research.^28^

Thermogravimetric analysis (TGA) was carried out on a Q5000 thermobalance (TA Instruments, USA) in a N_2_ atmosphere. The TGA test specimens were recovered from the crucible after the DSC tests. The heating rate was 10 °C/min, within the range of 50 to 600 °C. Each material was tested twice, and the average final residue value at 600 °C was used to estimate the PCL content, considering the weight loss exhibited by pristine GNP and rGO nanopapers.

Wide-angle X-ray scattering (WAXS) experiments on selected samples were performed with transmission geometry or grazing incidence (GIWAXS) at room temperature. These experiments were performed on a Xeuss 2.0 system (Xenocs SA), equipped with a microfocus Cu KαX-ray source (GeniX3D, 50 kV, 0.6 mA), which generates X-ray radiation with a wavelength of 1.5418 Å. The detector used was Pilatus 300K (DECTRIS, Switzerland) with a resolution of 487 × 619 pixels (pixel size = 172 × 172 μm^2^). The sample-to-detector distance was 138.61 mm, and the exposure time was 1800 s. The 1D intensity profiles were integrated from background-corrected 2D WAXS patterns within an azimuthal angle range of 0–90°.

Thermal diffusivity was measured by a xenon light flash apparatus (LFA, 467 HyperFlash by Netzsch) to obtain the in-plane thermal diffusivity (α) of the prepared nanopaper at 25 °C. Each specimen was tested 5 times (flash conditions: 180 V, 200 ms), and the average value of its thermal diffusivity was calculated using eq 2.

2 $$k=\rho \times \alpha \times C_{p}$$

where *k* is the thermal conductivity, ρ is the density of the nanopaper, and *C*_p_ is the specific heat capacity.

The specific heat capacity of nanopapers (*C*_p_) was calculated by the weighted average of *C*_p_ values of PCL at RT (2.0 J/gK)^61^ and graphite at RT (0.71 J/gK)^62^ for each sample using eq 3:

3 $$C_{p}=C_{pP}\times {Ø}_{PCL}+C_{pG}\times \left(1-{Ø}_{PCL}\right)$$

where $C_{pP}$ is the specific heat capacity of PCL, Ø_PCL_ is the weight percentage of PCL in the nanopapers (calculated from TGA), and the $C_{pG}$ is the specific heat capacity of graphite. Each sample’s density (ρ) was calculated by the mass and volume of five die-cut circular samples with a diameter of 5 mm taken from different positions on the nanopaper. For each disk, the thickness was measured with a micrometer gauge, while the mass was measured on the TGA balance (accuracy: 0.0001 mg) at RT, and the average values were calculated to determine the density.

## 3 Results and Discussion

### 3.1 Crystallization of PCL within GRM Nanopapers

#### 3.1.1 Effect of the GRM Structure and Polymer Content

Raman spectroscopy was employed to investigate the structural differences between GNP and rGO within the nanopapers prepared in this work. In particular, the intensity of the *D* band, centered at 1356 cm^–1^ and associated with graphene structural defects, and the *G* band, centered at 1580 cm^–1^, were analyzed. Typically, the intensity ratio (*I*_D_/*I*_G_) between the *D* and *G* bands is a parameter for quantifying structural disorder.^63–65^ This ratio is 0.08 for GNP, which is significantly lower than that for rGO, which is 0.85. This means that rGO possesses more structural defects, consistent with the harsher conditions applied for oxidation and subsequent thermal reduction. WAXS patterns for nanopapers obtained with GNP and rGO (Figure 1b) show the well-known strong (002) signal at 2θ = 26.5,^66,67^ which is significantly broader for rGO, according to its higher structural disorder. High-resolution C 1s X-ray photoelectron spectroscopy (XPS) measurements (Figure 1c,d) show that the sp^3^ carbon content is about 2.1% in GNP and about 2.3% in rGO. SEM micrographs of the nanopaper cross-sections (Figure 1e–h) evidence the microstructure of GNP and rGO. In particular, rGO nanopaper cross-sections exhibit wavy textures and significantly lower compactness than their GNP counterparts. This finding reflects the differences in the thermal production of GNP and rGO powders, resulting in different morphologies.^51^ This observation is consistent with their specific surface areas, which were measured at 30 and 117 m^2^/g (Figure S1) for GNP and rGO powders, respectively. Consequently, GNP nanopapers exhibited a higher compactness than their rGO counterparts, leading to significant differences in the thickness and porosity of the nanopapers, as shown in Figure 1e–h. It is worth mentioning that the surface area was measured at 19 and 70 m^2^/g for GNP and rGO nanopapers, respectively (Figure S1). This suggests that GNP or rGO flakes are self-assembling during filtration, producing relatively compact structures upon solvent removal. Finally, it is pointed out that no endothermic or exothermic peaks were observed in the DSC curves up to 200 °C for either GNP or rGO nanopapers (Figure S2), evidencing no phase transitions from GRM and negligible residual solvent.

**Figure 1:**
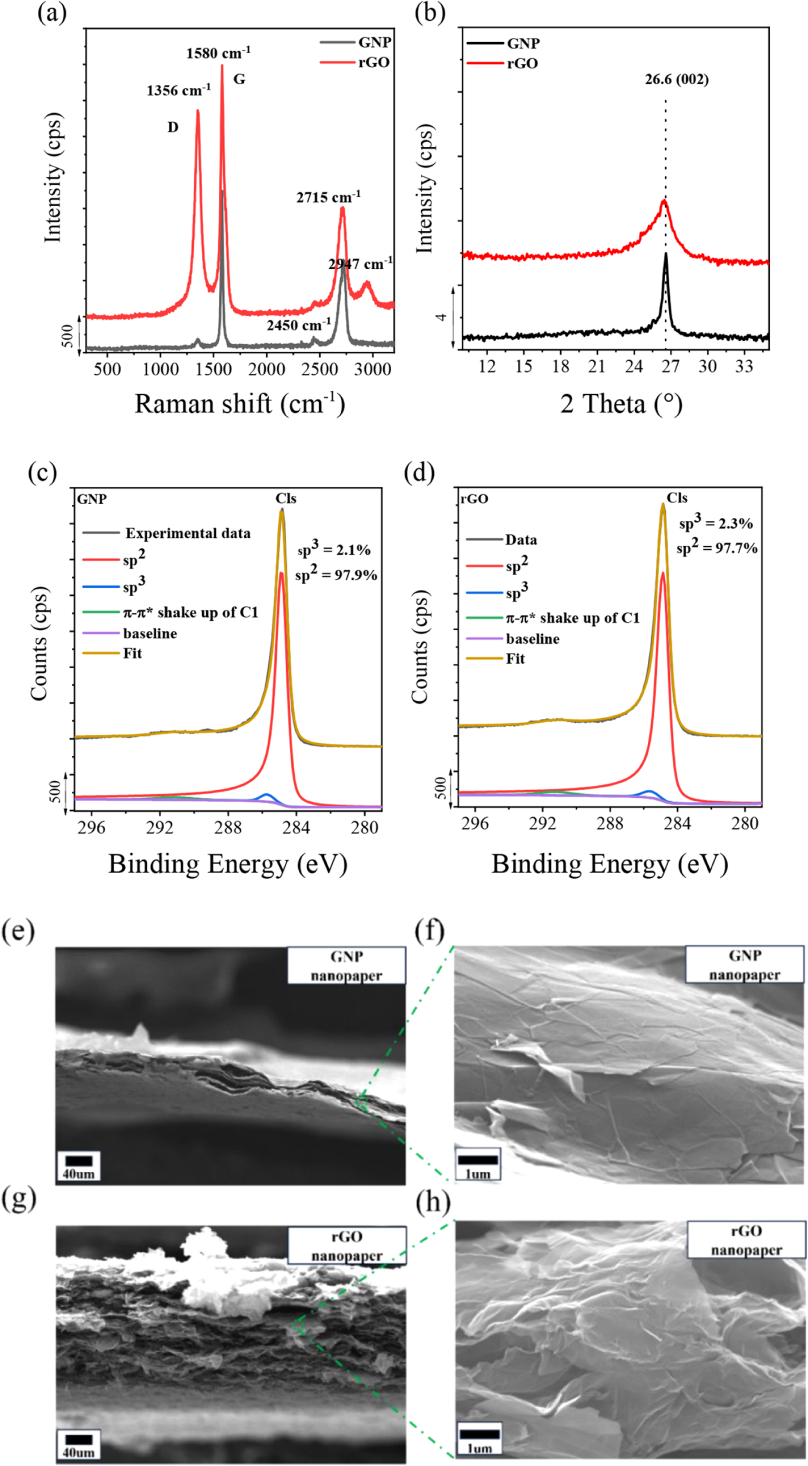
Comparison of GNP and rGO nanopapers: (a) Raman spectroscopy, (b) WAXS spectra, (c) C 1s XPS spectra of GNP, and (d) C 1s XPS spectra of rGO. SEM micrographs for a cross-section of nanopapers based on GNP (e, f) and rGO (g, h).

Table 1 shows the PCL content of samples prepared at different filtration times and the corresponding maximum decomposition rate temperatures (*DTG*_max_) obtained through TGA testing. TGA analysis of GRM/PCL nanopapers (Figure S3) showed no significant weight loss at temperatures below 300 °C, suggesting that a negligible amount of solvent is retained. Thus, the weight loss provides an estimation of the actual PCL content for each nanopaper. It is notable that *DTG*_max_ increases from 370 °C for pristine PCL to 407 and 410 °C for GNP and rGO nanopapers, respectively, which can be explained by the delayed release of low molecular weight products from PCL decomposition due to tortuous diffusion paths and/or absorption onto GRM flakes, in agreement with previous literature on polymer nanocomposites.^68^

This series of nanopapers with different PCL loadings and GRM features was systematically studied for its thermal properties and crystalline structure. For GNP-based nanopapers, the DSC heating scans (Figure 2a) for nanopapers containing different PCL loadings are consistent with previously reported results,^28^ displaying four distinct endothermic peaks, referred to as A, B, C, and D. In the DSC cooling scans, four crystallization exotherms during cooling from the melt (Figure S4) show a one-to-one correspondence with the four melting endotherms. These four endothermic peaks were previously assigned to the melting of unoriented PCL (A), oriented PCL (B), prefrozen PCL on the GNP surface (C), and the last peak (D), which was tentatively explained by the melting of prefrozen PCL within GNP galleries.^28^ With increasing PCL content, peak A becomes dominant, and the relative intensities of peaks B, C, and D steadily decrease (Table 2 and Figure S5). Eventually, when the PCL content exceeds 40%, the D peak becomes almost undetectable and negligible compared to the main crystalline fraction (A). rGO nanopapers display distinct calorimetric behavior, as shown in Figure 2b. At a PCL loading of 8.4%, i.e., the lowest level), only two endothermic peaks, A and D, can be observed. At a PCL loading of 20%, traces of the B and C signals appear. As the PCL content increases, the intensities and enthalpy values of peaks B, C, and D decrease (Table 2 and Figure S5). Only traces of the D peak can be detected when the PCL content exceeds 50%.

**Table 2:** PCL Content in Filtered Nanopapers by TGA and Enthalpies (Normalized by the Actual PCL Content in the Sample) for Endothermic Transitions during Second DSC Heating Scans^a^

			Δ*H* (J/g_PCL_) of the peaks from 2nd heating scan					
	PCL grade and content (wt %)		A	B	C	D	Total	Crystallinity *X*_c_ (%)
GNP/PCL	M2	2.5	n.d.	2.7	n.d.	1.8	4.5	3.2
	M4	4.0	0.4	8.9	<0.1	<0.1	10.7	7.7
	M10	3.5	1.1	4.9	0.6	1.1	7.7	5.5
	M50	4.7	6.7	9.1	3.0	1.0	19.8	14.2
	M50	15.7	39.3	3.6	1.1	0.5	44.5	31.9
	M50	27.7	51.6	2.3	0.7	<0.1	54.7	39.2
	M50	41.5	57.7	2.3	0.5	<0.1	60.6	43.4
	M50	79.4	73.4	0.6	0.2	<0.1	74.2	53.2
rGO/PCL	M2	2.0	n.d.	n.d.	n.d.	<0.1	<0.1	n.d
	M4	4.7	8.5	n.d.	n.d.	1.7	10.2	7.3
	M10	7.7	13.2	n.d.	n.d.	0.7	13.9	10.0
	M50	8.4	16.0	n.d.	n.d.	0.7	16.7	12.0
	M50	20.3	39.5	0.7	0.3	0.2	40.7	29.1
	M50	23.0	40.7	0.8	0.5	0.1	42.2	30.2
	M50	51.5	66.8	0.5	0.2	<0.1	67.4	48.3
	M50	61.6	72.1	0.4	0.1	<0.1	72.6	52.0

a n.d. is for non-detectable signals. The crystallinity degree (*X*_c_) was calculated by considering the total latent heat of fusion determined during the heating scans (i.e., the sum of all melting peak areas).

**Figure 2:**
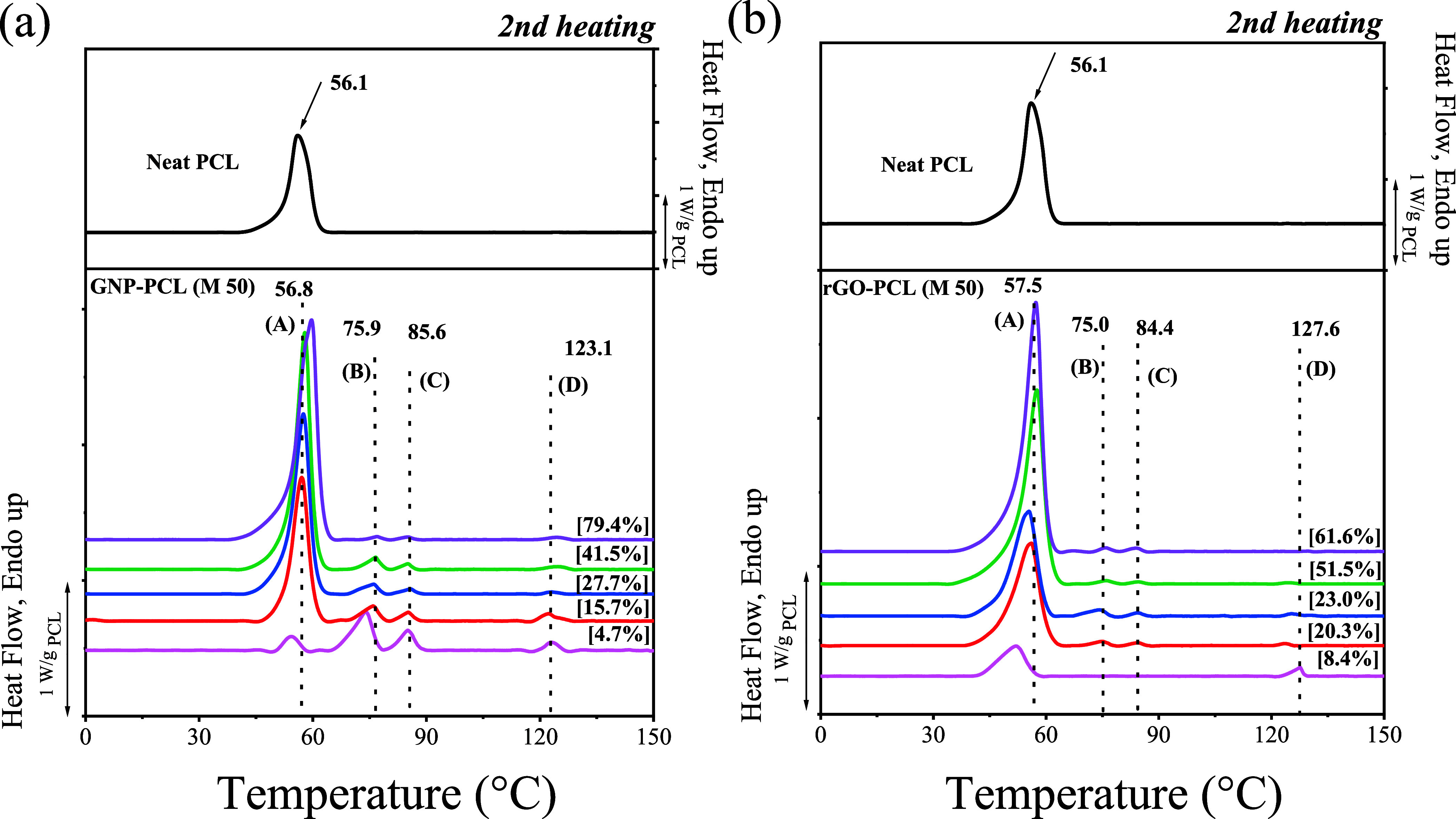
Second heating DSC curves of nanopapers with varying PCL content, prepared via filtration, for (a) GNP and (b) rGO. Plots are baseline subtracted and normalized by the actual PCL content, reported in square brackets. The raw data are reported in Figure S4.

These observations provide insights into the crystallization of PCL on the surface of GRM. At low PCL content, most of the polymer is strongly adsorbed onto the GRM flakes; therefore, the interaction between PCL and the surface appears to drive the crystallization of PCL. Conversely, a significant fraction of the polymer undergoes conventional crystallization at higher PCL content. Comparing the distinct calorimetric behavior of PCL on GNP and rGO, GNP yields a higher crystallization temperature (Figure S4) for the main peak (A), suggesting a higher nucleation efficiency. Furthermore, it is evident that the signals corresponding to the B, C, and D fractions are typically more pronounced in GNP-based nanopapers than in their rGO counterparts. This experimental evidence suggests that greater defectiveness, either in terms of 2D lattice imperfections (e.g., vacancies, residual oxidation, etc.) or more crumpled topography on the surface of rGO, limits the activity of GRM in polymer nucleation as well as in the formation of oriented and prefrozen crystals.

Concerning the total crystallinity, at a relatively high PCL content, the crystallinity closely resembles that of pristine PCL, within experimental errors and under the simplified calculation of total enthalpy as the sum of all endothermic peak integrals. Conversely, at a lower PCL content, the intricate organization of PCL and GRM induces a strong decrease in crystallinity. This suggests that the adsorption of PCL onto GRM limits the mobility of the polymer chains, and only a limited fraction of the polymer can effectively organize into crystalline domains. This observation supports a complex interplay between different factors, including prefreezing, heterogeneous nucleation, chain orientation, and restricted crystallization.

To further investigate the adsorption of PCL on GRM and its correlation with the DSC signals, different nanopapers were prepared by varying the sonication time to promote partial exfoliation of the GRM and, therefore, increase the polymer/GRM interfacial area in nanopapers. Indeed, increasing the sonication time from 1 to 5 h leads to much higher PCL contents in the nanopapers, at ca. 21.8% and 17.9% for GNP and rGO, respectively (Figure S7). However, the relative intensities of signals associated with the B, C, and D melting peaks (Figure 3) are not constant with the sonication time. In fact, the fraction of crystals corresponding to peaks B, C, and D decreases, so most of the polymer retained in the nanopapers prepared after 5 h sonication results in unoriented crystals. These DSC profiles reflect those for nanopapers with the corresponding PCL contents (Figure 2). This suggests that the higher PCL content is related to higher solution retention during filtration rather than higher polymer adsorption onto GRM flakes due to better dispersion and/or partial exfoliation during the longer sonication time. It is also worth mentioning that the longer sonication time slightly increases the defectiveness of the flakes, as observed by SEM (Figure S8) and Raman spectra (Figure S9). Furthermore, selected nanopapers underwent PCL extraction in toluene, which was previously reported to remove A, B, and C crystalline populations while retaining the D population.^28^ Indeed, Soxhlet extraction removes most of the PCL but not all of it, yielding a polymer content of about 3% (Figure S7) after the extraction for both GNP and rGO, regardless of the sonication time. The impossibility of removing part of PCL may be explained by a strong interaction of PCL with GRM flakes and/or the polymer confinement into domains practically inaccessible to the solvent. However, the amount of retained polymer does not appear dependent on the polymer/GRM interfacial area, suggesting the origin of D peak crystals is not a simple surface phenomenon. The remaining PCL is associated with a clear endothermic signal at ca. 130 °C for both GNP- and rGO-based nanopapers, regardless of the sonication time. Furthermore, compared to the nanopapers before extraction, the intensity and the temperature (+9 °C) for the D peak significantly increase. This point is particularly interesting as it suggests that the solvent treatment induces some reorganization of the crystals. While toluene cannot solubilize this PCL fraction, it appears to promote the reorganization or growth of crystals responsible for the D peak.

**Figure 3:**
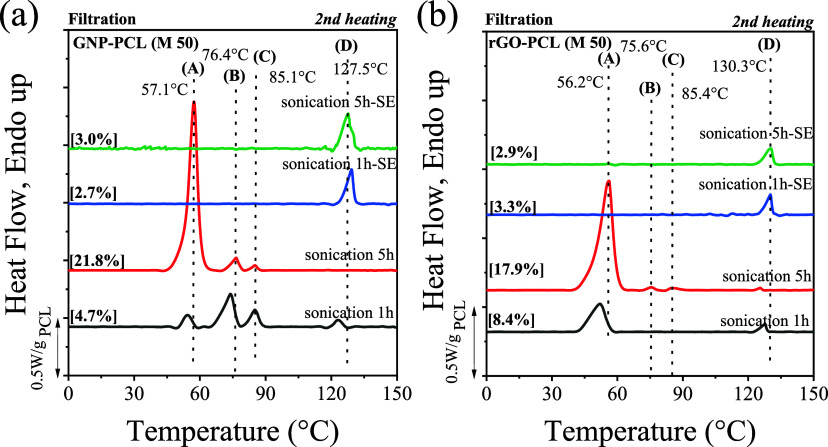
DSC second heating curves for selected nanopapers preparation via the filtration method based on GNP (a) and rGO (b). SE identifies Soxhlet-extracted nanopapers. Plots are baseline subtracted and normalized by the actual PCL content, reported in square brackets. Raw data are reported in Figure S6.

#### 3.1.2 Effect of Polymer Molecular Weight

It is well-known that the molecular weight of semicrystalline polymers plays a crucial role in influencing crystallinity (*X*_c_), melting temperature (*T*_m_), and crystal morphology.^69^ In the case of PCL, as the molecular weight increases, *T*_m_ also increases and eventually stabilizes, reaching a plateau above a certain molecular weight (approximately *M*_n_ = 10 kg/mol).^69^ In this regard, we investigated the crystallization behavior of PCL with different molecular weights within GRM nanopapers. Indeed, the crystallinity of the four neat PCL samples, characterized by different molecular weights determined from the second DSC heating curves (Figure S10), is 47, 57, 67, and 75%, for M50, M10, M4, and M2, respectively. A similar qualitative trend has been found in a recent report, where the *X*_c_ for PCL samples as a function of *M*_n_ first increased, reaching a peak value at 2 kg/mol, and then decreased as the *M*_n_ value increased, this trend being explained by a competition between secondary nucleation and diffusion.^69^

DSC curves (Figure 4) for GNP or rGO nanopapers containing a low fraction of PCL with different molecular weights are qualitatively similar, confirming the multiple melting peaks with the presence of the high thermal stability fraction referred to as D. However, with the decrease in PCL molecular weight, the total enthalpy value decreases (Table 2). This is unexpected based on the higher crystallinity exhibited by lower molecular weight PCL.^69^ This suggests that the mobility of short polymer chains may be strongly reduced once they are adsorbed onto the GRM, thus limiting their crystallization. In particular, for both GNP and rGO, the A melting peak becomes almost undetectable at a molecular weight of 2000 g/mol. This phenomenon underscores that at this low molecular weight, chain segments experience stringent constraints, preventing their crystallization in conventional crystals (A peak). It should be noted that peak A corresponds to the usual melting point of neat bulk PCL or the melting of PCL crystals that are similar to those in bulk, i.e., crystals where the chains do not interact with the filler and are also not confined within the intricate structure of the nanopapers. Any decrease in the enthalpy value of melting peak A can be interpreted as being due to the reduced chain diffusion caused by interactions between the chains and the GNP or rGO, or even confinement effects.

**Figure 4:**
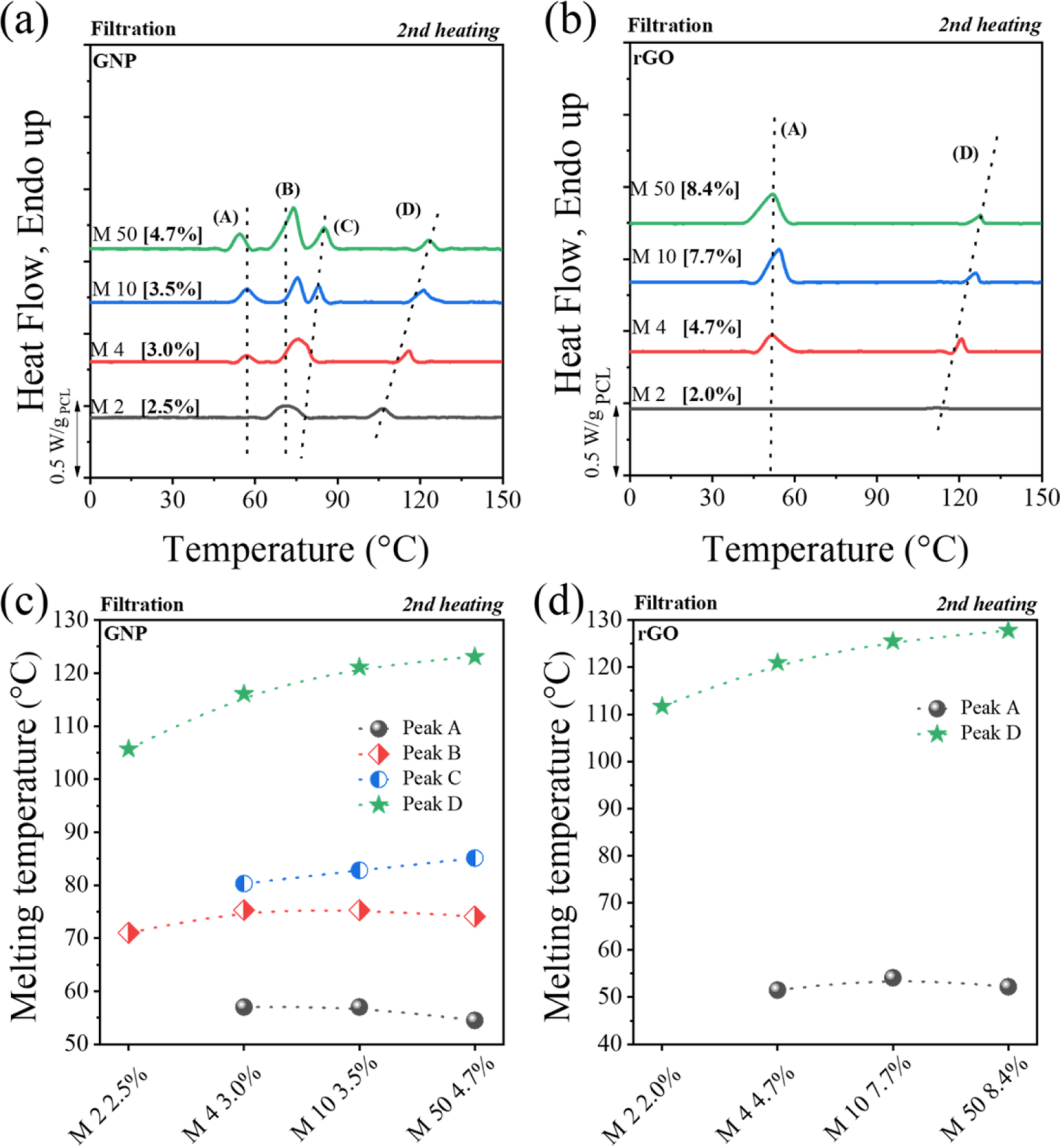
DSC second heating curves for different molecular weight PCL nanopaper preparation via the filtration method: GNP (a) and rGO (b). Plots are baseline subtracted and normalized by the actual PCL content, reported in square brackets. Raw data are reported in Figure S11. The relationship between the temperature of the different endo peaks and the molecular weight of PCL for nanopapers was based on GNP (c) and rGO (d).

The equilibrium melting point (*T*_m_^0^) of PCL crystals also depends on the *M*_n_ value, but it is in the range of 62 °C (for 2 kg/mol) to 75–80 °C (for *M*_n_ values between 4 and 70 kg/mol).^69^ *T*_m_^0^ is a theoretical value (usually obtained by extrapolation of experimental data), which indicates the melting point of lamellar crystals with an extended chain conformation (or infinite crystals with no surfaces) corresponding to the maximum thermodynamic stability. Therefore, in the case of the nanopapers prepared here, the experimentally found peak A has a melting point below *T*_m_^0^, thus corresponding to the unrestricted melting of PCL crystals, which behave in the same way as neat PCL bulk crystals. Peak B has a melting point just below *T*_m_^0^ (which could correspond to the melting of chain-oriented crystals, see below), and peaks C and D have experimentally determined melting peaks with much higher values than *T*_m_^0^ (i.e., 85 °C for the C peak and 120–130 °C for the D peak). These values higher than the equilibrium melting point can be explained only by assuming crystals are strongly adsorbed on the GNP or rGO, leading to a particularly stable structure that can only disappear by heating well above *T*_m_^0^. Notably, the decrease in molecular weight has no strong effect on peak D intensity, as the change in the enthalpy values for that signal is not significant (Table 2 and Figure S12). However, with the reduction in molecular weight, the melting point of peak D shifts toward lower temperatures (Figure 4c,d) for both GNP and rGO nanopapers. A similar trend is observed for the melting point of the C peak (only visible in GNP nanopapers), eventually causing a partial overlap with the B peak at lower molecular weight. In contrast, A and B peak temperatures are independent of the PCL molecular weight. This suggests that the molecular weight affects the stability of the crystals that melt in peaks C and D. As the existence of such ordered structures above the PCL melting point is associated with strong interfacial interactions, it appears that longer chains yield stronger adsorption onto the GRM. Further insight may be obtained from the thermodynamic model proposed for prefrozen layers, known to be stable at temperatures above $T_{m}^{0}$ . Their formation was initially described based on the interfacial free energy between the polymer (melt or crystal) and the substrate, according to Young’s equation^45^ (eq 4):

4 $$\gamma_{sub,liq=}\gamma_{sub,cry}+\gamma_{cry,liq}\times \cos\theta$$

In brief, when $\gamma_{sub,liq}>\gamma_{sub,cry}+\gamma_{cry,liq}$ , the contact angle θ equals zero, and the crystalline phase wets the surface. A comprehensive thermodynamic theory for polymer prefreezing was later formulated by Thurn-Albrecht et al.,^70^ computing the free energy change as a function of thickness and temperature, Δ∑(*l*,*T*), upon formation of a prefrozen layer from the melt on a substrate, according to eq 5:

5 $$\Delta \Sigma \left(l,T\right)=\gamma_{sub,cry}+\gamma_{cry,liq}-\gamma_{sub,liq}+\Delta S\times l\times \left(T-T_{m}^{0}\right)\times \frac{T}{T_{m}^{0}}+\gamma_{sub,liq}\times \exp\left(-\frac{l}{l_{o}}\right)$$

The three terms in this equation represent 1) the surface energy balance $\gamma_{sub,cry}+\gamma_{cry,liq}-\gamma_{sub,liq}$ , 2) the entropic term associated with bulk entropy change during crystallization Δ*S*, and 3) the interaction between the substrate and melt, separated by the prefrozen layer, decaying with the correlation length *l*_o_. The maximum thermal stability (*T*_max_) of the prefrozen layer was also derived as follows (eq 6):

6 $$T_{max}=\frac{T_{m}^{0}}{2}\left[1+\sqrt{1+\frac{4}{\Delta S\times l_{o}\times T_{m}^{0}}\times \frac{\Delta \gamma }{1+\Gamma^{-1}\left(2,\frac{\Delta \gamma }{\gamma_{sub,liq}}\right)}}\right]$$

where $\Delta \gamma =\gamma_{sub,liq}-\left(\gamma_{sub,cry}+\gamma_{cry,liq}\right)$ .

Changing the molecular weight of the polymer chains may affect both surface energies and bulk entropies, taking into account the arrangement of molecules on the liquid and in crystals, which in turn affects both Δγ and Δ*S*. While a quantitative study of the effect of molecular weight on thermodynamics parameters is beyond the scope of this paper, this model appears to support the change in thermal stability of prefrozen crystals with the PCL molecular weight.

Crystallization temperatures (Figure S11) follow the same trend as that observed for the function of *M*_n_. It is interesting to note how the crystallization exotherms corresponding to peaks C (*T*_c_ values of 65 and 75 °C) and D (*T*_c_ values of 95 and 105 °C) occur at significantly higher temperatures compared to the crystallization temperature of isotropic PCL bulk samples (typically 25–30 °C). These elevated crystallization temperatures can also be explained by the very high melting temperatures, which correspond to the higher stability crystals formed in peaks C and D (Figure S11).

#### 3.1.3 Effect of the Preparation Method

Nanopapers were prepared using various procedures to examine the role of the solvent in the interaction of PCL with GRM. Indeed, the dispersion of GRM by sonication is effective in a limited number of solvents, most of which are difficult to remove due to their high boiling points or are not suitable solvents for PCL. Therefore, the impregnation of preformed GRM nanopapers with different polymer solutions was exploited to investigate the effect of different solvents within nanopapers having the same structural features. In particular, pristine GRM nanopapers were produced by filtration of GRM suspensions in DMF, followed by impregnation with PCL solution in either DMF or toluene, to obtain nanopapers with comparable polymer content. Crystallization of nanopapers prepared by impregnation with PCL solutions was studied in comparison with counterparts obtained by filtration. For GNP, nanopapers prepared through different methods exhibit qualitatively similar DSC curves (Figure 5a), with clear traces of A, B, and C peaks in all cases. Regarding the D peak signal, it is clearly observable in the nanopaper impregnated with the PCL DMF solution, whereas impregnation from the toluene solution does not appear to produce a significant fraction of D crystals. Furthermore, the enthalpy values of the four melting peaks (Figure 5c) indicate that the nanosheets obtained by the impregnation method have a lower fraction of highly stable crystals, particularly pronounced when using toluene as the solvent. For rGO nanopapers, the DSC curve and melting peak enthalpy (see Figure 5b,d) of nanopapers obtained by the impregnation method show signals related only to unoriented type A PCL crystals, without visible high-temperature melting peaks B, C, and D. For B and C peaks, this is consistent with the findings on the corresponding rGO nanopaper obtained by filtration, thus confirming that rGO is not effective in triggering the formation of such crystal populations.

**Figure 5:**
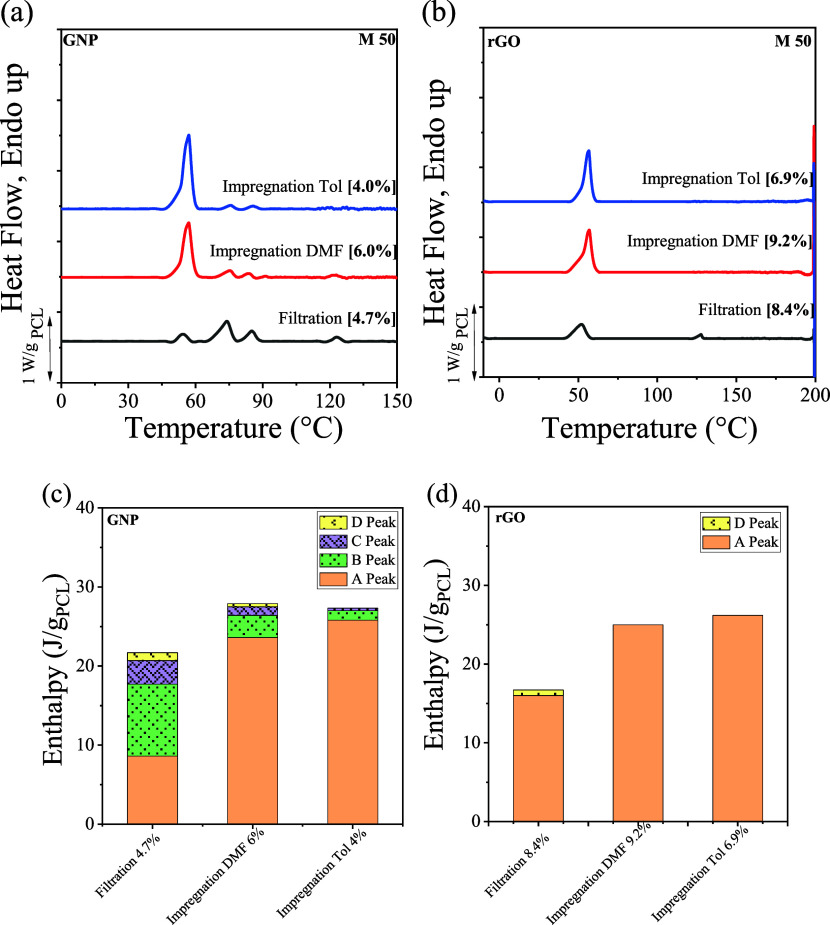
DSC second heating curves (obtained immediately after cooling from the melt) for different preparation methods and solvents for GNP (a) and rGO (b). Plots are baseline subtracted and normalized by the actual PCL content, reported in square brackets. Raw data are reported in Figures S14 and S15. Integral enthalpy values of the endothermic peaks obtained during the second DSC heating scans for the different nanopapers, as a function of different preparation methods and solvents for GNP (c) and rGO (d).

Therefore, the impregnation method appears to be less effective for the organization of highly stable crystals. This could be related to the different durations allowed for the polymer to diffuse, adsorb, and self-organize on the surface of GRM plates, which in this method is controlled by the solvent evaporation rate. Given the higher volatility of toluene compared to DMF, this may also explain why impregnation from toluene solution is the least effective method to produce highly stable PCL crystal fractions. Overall, it appears that the sonication of GRM particles in the presence of dissolved polymer plays an essential role in controlling the adsorption of PCL onto GNP/rGO flakes, with possible consequences on the macromolecules’ mobility, in turn affecting their crystalline organization.

### 3.2 Structural Investigation of the Crystalline Fractions

To further investigate the origin and properties of the different crystalline fractions observed and discussed above, selected nanopapers were studied by the DSC-based successive self-nucleation and annealing (SSA) thermal fractionation method and by X-ray diffraction.

#### 3.2.1 Successive Self-Nucleation and Annealing

The SSA technique can fractionate samples by a carefully designed thermal protocol.^58–60^ After the SSA protocol is applied, the final DSC heating scans show multiple melting peaks produced by successive molecular segregation, self-nucleation, and annealing. The melting peak distribution is proportional to the distribution of the lamellar thicknesses produced by thermal fractionation. Each peak corresponds to the melting of a thermal fraction. The higher the melting point of the fraction, the thicker the average lamellar crystals that melt in it. This melting point distribution can be generated by differences in molecular weights, particularly in neat materials, differences in the distribution of defects or phases in copolymers, and, depending on the filler/nanoparticle content and specific interactions, by differences in the ways of assembly.^71–77^ In this work, as in our previous work,^28^ we found different levels of organization due to various processes induced by the nanoplates. However, in this case, other factors, such as the type of nanoplate and the molecular weight, also play a key role, which we discuss below.

Figure 6 compares PCL’s SSA final heating curves obtained from GNP and rGO nanopapers and investigates differences in molecular weight (M50 and M10), preparation methods, and the variance in impregnation solvents.

**Figure 6:**
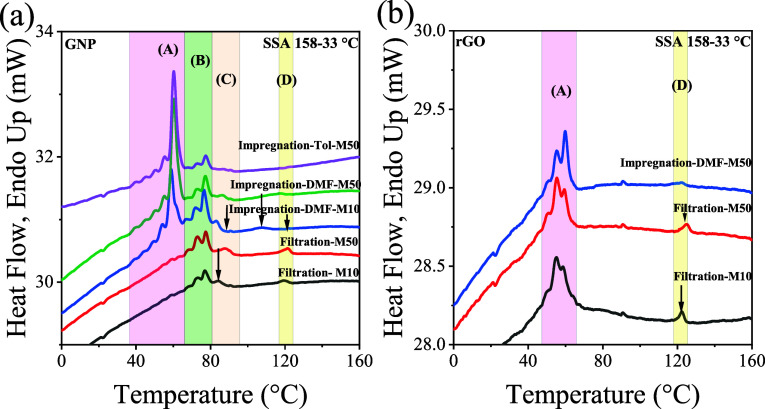
SSA final DSC heating scans for different molecular weights and different preparation methods and solvents of nanopapers for the (a) GNP and (b) rGO nanopapers.

Figure 6a shows that the SSA on PCL embedded within GNP-based nanopapers displays a series of melting fractions that can be grouped into four different melting ranges (shown in the figure by different vertical shadowed regions, as peaks A to D) corresponding to the four melting peaks obtained by standard DSC nonisothermal tests. It should be noted that the thermal fractions within zone A melt at temperatures similar to those in SSA-fractionated bulk PCL. The melting peaks in zone B are already in a temperature range similar to that of *T*_m_^0^. Peaks corresponding to C and D melt at higher temperatures than *T*_m_^0^, similar to the results obtained by standard nonisothermal DSC. However, differences in the relative intensities arise between nanopapers prepared by filtration or impregnation methods and molecular weight, reflecting and confirming the above results for conventional nonisothermal DSC results. In particular, the fractions that melt in zone A (Figure 6a) are almost undetectable in nanopapers obtained by filtration, whereas they become the main endothermic signals in impregnated nanopapers.

Minor differences are observable when comparing results for different PCL molecular weights (M10 vs M50), whereas the role of the solvent used for impregnation is crucial (see Figure 6a and compare with neat PCL thermal fractionation results in Figure S16). Indeed, impregnation from a toluene solution of PCL was found to display only peak ranges A and B, whereas traces of peaks C and D are found from impregnation from a DMF solution, yet with lower intensity compared to the corresponding peaks in nanopapers prepared by filtration. Furthermore, at the same molecular weight, the temperature range of peaks C and D is lower (e.g., for M50, ca. 1.8 and 4.0 °C for C and D, respectively) in the nanopapers prepared by impregnation compared to those obtained by filtration, suggesting a weaker interaction is achieved by impregnation.

The added value from SSA analysis is that we can obtain insight into the origin of the melting peaks, depending on their fractionation capacity. Peaks A and B observed in GNP nanopapers can be thermally fractionated by SSA. Peak A positions and fractionation are similar to those obtained in bulk neat PCL (see Figure S16); thus, peak A in the conventional DSC results (or peaks in zone A after SSA) corresponds to the melting of unoriented PCL crystals. Peaks B in Figure 6a are observed at temperatures higher than those obtained by fractionating the standard PCL (Figure S16). This denotes that graphene can induce certain crystal orientation levels, and thus, peak B corresponds to the melting of oriented PCL crystals. Peak C occurs at even higher temperatures, even above the equilibrium melting point of PCL (*T*_m_^0^), and is consistent with the findings of prefreezing phenomena previously reported by Thurn-Albrecht et al.^43–45,78^ Interestingly, melting peak C remains unfractionated despite the application of the SSA protocol, evidencing that this peak has a different origin, which appears compatible with a prefreezing phenomenon on the surface of GRM flakes. Indeed, while a certain chain mobility is required during SSA to anneal and perfect the crystals by thickening, the polymer chains prefrozen in a crystalline layer and strongly bound onto GRM may not diffuse, and therefore, the crystals corresponding to peak C cannot anneal. Similarly, peak D is also unable to be fractionated by SSA, confirming our previous report.^28^ Even when the lack of thermal fractionation cannot explain the exact origins of peaks C and D, their systematic presence demonstrates the existence of highly stable PCL-oriented crystals due to strong interactions between PCL and the GNP flakes.

In rGO-PCL nanopapers, only peaks A and D are detectable after SSA, which agrees with results obtained during conventional nonisothermal DSC tests, where neither the crystallization exotherms nor the melting endotherms corresponding to peaks B and C were observed (see Figure S11). One hypothesis for the absence of peaks B and C is that the structural defects on rGO negatively affect the nucleation capacity of the rGO versus PCL and the transcrystallinity reported for the prefreezing phenomena. For this reason, the PCL is not highly oriented by the rGO action or does not experience the prefreezing phenomena, which is in clear contrast to the case of GNP. Nevertheless, peak D is present independently of the type of graphene. Interestingly, peak D in rGO-PCL nanopapers obtained by filtration appears stronger than the corresponding signal in the GNP nanopapers. Considering the higher surface area for rGO compared to that for GNP, it is reasonable to assume that the higher interfacial surface with the PCL in solution may lead to a higher PCL adsorption onto dispersed particles. Once deposited and organized in the nanopapers by filtration and subsequent drying, the adsorbed PCL is probably trapped between rGO layers, leading to the peculiar organization reflected by peak D.

#### 3.2.2 X-ray Diffraction

X-ray diffraction analysis revealed characteristics of both PCL and graphite components in the nanopaper composites. The PCL phase exhibited characteristic diffraction peaks at 2θ = 21.4°, 22.0°, and 23.7° (Figure S17), corresponding to the (110), (111), and (200) planes of its orthorhombic crystal structure, respectively. WAXS patterns in Figure 7 demonstrated the crystal signals of PCL and the (002) peak of graphite.

**Figure 7:**
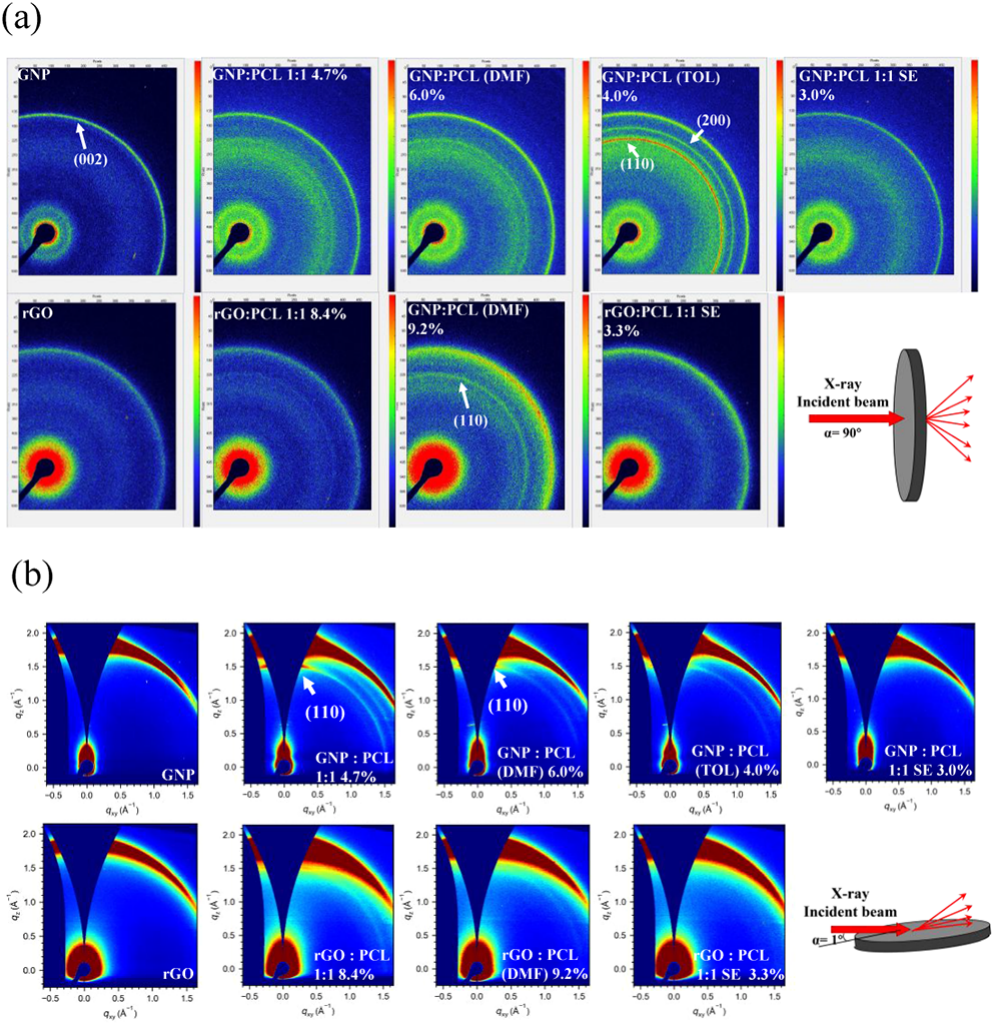
2D WAXS patterns of a series of nanopapers (a) in transmission mode and (b) in GIWAXS mode.

Two experimental configurations were selected to investigate the crystallographic orientation of the PCL crystals in the nanopapers. The transmission mode analysis (Figure 7a) reveals no preferred orientation of the PCL crystallites. However, notable intensity differences were observed between filtration-derived and impregnation-prepared nanopapers. As shown in Figure S18, the impregnation method yielded stronger signals from PCL crystallites than those prepared by filtration. This divergence correlates with reduced interfacial interactions between PCL and GRM during filtration, as previously evidenced by DSC analysis (Figure 5), resulting in fewer crystallization constraints. In particular, the diffusion of PCL chains within the interlayer gaps and pores of the nanopapers was hindered during the impregnation process due to high solution viscosity and limited processing time caused by rapid solvent evaporation. Furthermore, no difference in PCL peak positions was observed, indicating no alterations in the crystalline form of PCL. Filtration-derived nanopapers were Soxhlet-extracted to remove dissolvable components. WAXS of the extracted nanopapers exhibits no clear PCL crystal signals. This could originate from two possible reasons: (i) the low weight fraction of PCL in the extracted nanopapers or (ii) the decrease in the crystallinity of PCL in close vicinity to the surface of GNP. This causes an insufficient signal-to-noise ratio of the PCL crystal signals. Thus, the current results do not allow a conclusive interpretation of the nature of the transitions in the DSC even after PCL extraction (Figure 3).

The GIWAXS mode analysis (Figure 7b) revealed a pronounced preferred orientation of the PCL (110) plane, exhibiting a potential correlation with the orientation of the oriented (002) graphite plane. The degree of orientation of the (002) plane can be determined from the azimuthal distribution profile, as shown in Figure S19a, which is higher in rGO nanopapers than in rGO nanopapers. This hierarchy in graphitic ordering was consistent with the observations from cross-sectional SEM imaging (Figure 1). Furthermore, GNP-based composites exhibited enhanced PCL (110) orientation relative to rGO systems (Figure S19b,c), suggesting that the orientation of PCL depends on the orientation of graphitic flakes.

### 3.3 Thermal Conductivity of Nanopapers

The thermal conductivity of nanopapers depends on the interplay between nanoparticle conductivity and the efficiency of thermal contacts. In the presence of a polymer binder, the crystallinity of the polymer is an important parameter for controlling heat spread across conductive nanopapers. In particular, the crystalline structure of a polymer layer between conductive particles may affect the efficiency of thermal contacts.^28^ With the aim of investigating the correlation between the morphology of GRM, PCL content, and crystallinity, thermal conductivity was systematically studied for different nanopapers. Thermal diffusivity values and densities measured (Table S3) for pristine GRM nanopapers show their high thermal diffusivity and relatively low density, owing to their porosity. The thermal conductivity of pristine GNP was calculated to be approximately 108 W/m·K. For rGO, a much lower value of 8.6 W/m·K was obtained due to both its significantly lower density and higher structural defectiveness compared to GNP. On the one hand, the presence of PCL typically yielded an increase in the density of nanopapers. This is explained by the adhesive role of the polymer between GRM flakes, which enhances the packing of flakes and reduces porosity, as observed by SEM on nanopaper cross-sections (Figure S20). On the other hand, PCL generally decreased thermal diffusivity as a function of polymer content. The effects of density and thermal diffusivity were computed in the calculation of thermal conductivity, which was typically higher for nanopapers containing PCL compared to pristine GRM nanopapers, evidencing that the effect of enhanced density dominates over the limited reduction in thermal diffusivity (Figure S21). Overall, nanopapers based on GNP exhibit thermal conductivities in the range of 125 and 161 W/m·K, whereas rGO nanopapers range from 7.1 to 11.7 W/m·K, with lower-end values reflecting the poor density and inhomogeneity obtained by the infiltration process. Unfortunately, considering the large experimental errors in both density and diffusivity values and their propagation to the thermal conductivity values (Table S3 and Figure S21), limited differences in heat transfer performance can be claimed between the different formulations of nanopapers as a function of PCL molecular weight. In this scenario, a possible role of the PCL multiple crystalline populations on phonon transmission at particle–particle contacts remains elusive and will require further studies.

## 4 Conclusions

Thermally conductive nanopapers based on graphite nanoplates and reduced graphene oxide were prepared in the presence of polycaprolactone. The peculiar crystallization of PCL in multiple crystalline populations within such nanopapers was studied in detail. Conventional PCL crystals (peak A), corresponding to the same melting temperature as pristine PCL, represent the main signal only in nanopapers containing a high PCL content. When the polymer content is decreased, the relative intensity of peak A becomes progressively lower, indicating that most of the PCL is strongly influenced by the interactions with the GRM flakes. The effects of this interaction include higher stability crystals obtained as a result of the well-known strong nucleation onto GRM (peak B), as well as two other signals (peaks C and D) at temperatures above the equilibrium melting temperature for PCL, apparently related to prewetting crystalline layers. The relative intensities of the different signals were found to be strongly dependent on the structure of GRM flakes, the PCL molecular weight, and the preparation procedure.

The comparison between GNP and rGO provided insights into the correlation between the PCL crystalline populations and the structural features of GRM. Indeed, the plentiful structural defects remaining on rGO after its thermal reduction, including bending, sp^3^ carbons, and vacancies, can be associated with very low intensities of peaks B and C. Interestingly, peak D is clearly observable in both GNP and rGO nanopapers at low PCL content, and this is the only signal retained after the extraction of PCL in Soxhlet. The impossibility of dissolving part of PCL in toluene demonstrates that either the strength of PCL adsorption dominates the free energy of dissolution or the remaining amount of PCL is inaccessible to the solvent. The latter explanation appears fascinating, as it may correspond to thin layers of PCL within galleries or slit pores in the expanded GRM.

Further insight into the origin of the C and D peaks was obtained by successive self-nucleation and annealing studies, which evidenced that both peaks cannot be fractionated, as they are strongly adsorbed crystalline layers in which molecules cannot diffuse, thereby preventing crystal annealing. Peaks C and D are therefore compatible with the presence of a prefrozen crystal layer, which requires temperatures higher than the equilibrium melting temperature of PCL to desorb and subsequently melt.

Concerning the processing methods of nanopapers, comparable results were obtained by the conventional filtration of GRM suspension in a diluted polymer solution or by the impregnation of preformed GRM nanopapers with a solution of PCL. Indeed, small DSC signals corresponding to the B, C, and D melting peaks were observed in impregnated GNP nanopapers, but with lower relative intensities than the corresponding nanopapers obtained by filtration. This is likely explained by the reduced time allowed for PCL adsorption and organization on the inorganic surfaces due to the higher solution viscosity and the limited time available caused by the simultaneous evaporation of the solvent.

Concerning the heat transfer performance of the proposed nanopapers, PCL was confirmed as an efficient binder for GRM flakes, leading to a denser assembly of nanoflakes within the nanopaper. This enhancement in density was found to dominate over the limited reduction in thermal diffusivity caused by the introduction of a poorly conductive polymer. Overall, the thermal conductivity of composite GRM/PCL nanopapers, reaching up to 161 W/m·K, outperformed the conductivity of pristine GRM reference nanopapers prepared under the same conditions. Therefore, organic/inorganic nanopapers appear to be a promising solution for obtaining flexible heat spreaders for electronics and other low-temperature heat management applications.

## Supplementary Material



## Notes

Additional characterization of nanoparticles and nanopapers; N_2_ adsorption isotherms and pore size distribution, DSC, TGA, SEM, Raman, WAXS, and thermal conductivity (PDF)
