# Supplementary material for: Crystallization of Polycaprolactone within Nanopapers Based on Graphene-Related Materials
Source: Macromolecules. 2025 Jun 30;58(14):7343–57. doi: 10.1021/acs.macromol.5c00752 (PMC12288078; doi:10.1021/acs.macromol.5c00752)
Supplement: Supplementary file 1 [file ma5c00752_si_001.pdf]

Supporting information

Crystallization of polycaprolactone within nanopapers based on graphene-related materials

Hui Zhao[1], Ricardo A. Pérez-Camargo[2], Giacomo Damonte[3], Marco Armandi[4], Orietta Monticelli[3], Guoming Liu[5,6], Alejandro J. Müller*[2,7] and Alberto Fina*[1]

[1] Dipartimento di Scienza Applicata e Tecnologia, Politecnico di Torino- Alessandria campus, viale Teresa Michel, 5, 15121 Alessandria, Italy

[2] POLYMAT and Department of Polymers and Advanced Materials: Physics, Chemistry and Technology, Faculty of Chemistry, University of the Basque Country UPV/EHU, Paseo Manuel de Lardizabal 3, 20018, Donostia-San Sebastián, Spain

[3] Dipartimento di Chimica e Chimica Industriale, Università di Genova, Via Dodecaneso 31, 16146 Genova, Italy

[4] Dipartimento di Scienza Applicata e Tecnologia, Politecnico di Torino, Corso Duca degli Abruzzi 24, 10129 Torino, Italy

[5]Beijing National Laboratory for Molecular Sciences, Institute of Chemistry, Chinese Academy of Sciences, Beijing 100190, China

[6] University of Chinese Academy of Sciences, Beijing 100049, China

[7] IKERBASQUE, Basque Foundation for Science, Bilbao, 48009, Spain

*Corresponding authors: alejandrojesus.muller@ehu.es; alberto.fina@polito.it

Table S1. Nanopapers list, with codes and preparation conditions (Filtration).

Filtration time (h)	Sonicatio n time	Ratio GNP : PCL in suspension	Filter Diame ter [mm]	Pressing method	PCL molec ular weight
5	5s on and 5s off for 0.5h	1:1	47	RT	M50
		1:5	47	RT	
		1:1	47	RT	
		1:5	47	RT	
17		1:1	47	RT	
		1:5	47	RT	
		1:1	47	RT	
		1:5	47	RT	
72		1:1	47	RT	
		1:1	47	RT	
		1:1	47	RT	M10
		1:1	47	RT	
		1:1	47	RT	M4
		1:1	47	RT	
		1:1	47	RT	M2
		1:1	47	RT	
	5s on and 5s off for 2.5h	1:1	47	RT	M50
		1:1	47	RT	

Table S2. Nanopapers list, with codes and preparation conditions (M50, Imprgnation).

Sample code	Ratio GNP:PCL in suspension	Solvent	Pressing method
GNP:PCL	10:1	DMF	RT
rGO:PCL			
GNP:PCL		Toluene	
rGO:PCL			

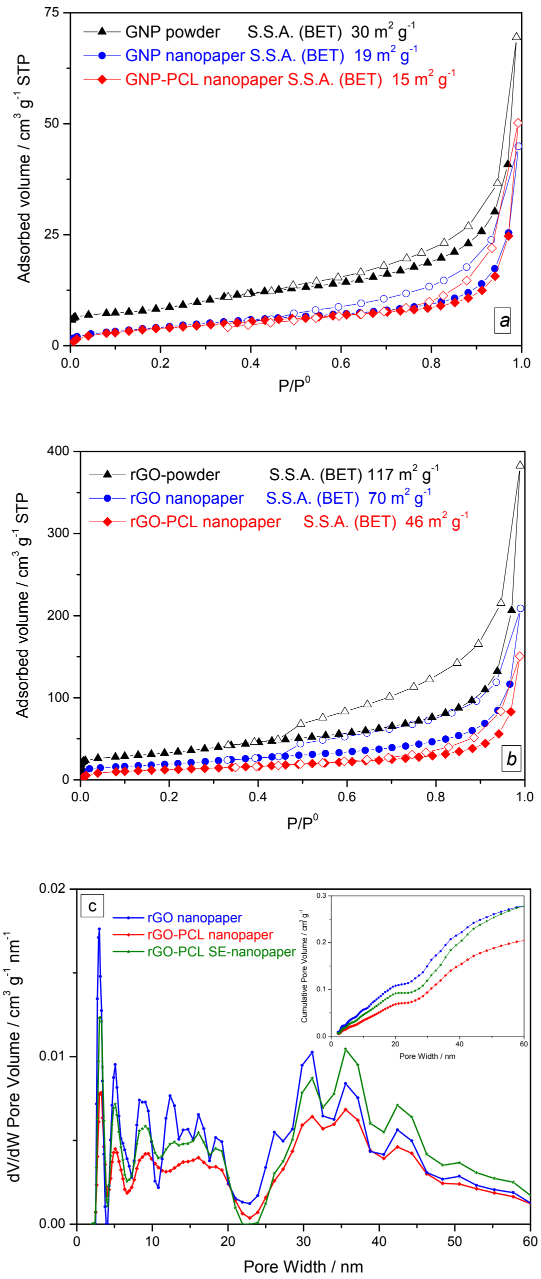

Figure S1. N_2 adsorption isotherms at 77 K on GNP- (section a) and rGO-based materials (section b). Pore size distribution as calculated by NLDFT method for rGO nanopaper (blue), rGO:PCL 1:1 (red), and rGO:PCL 1:1 after toluene Soxhlet extraction (SE) of PCL (section c). The inset of section c shows the corresponding integrated cumulative pore volume curves.

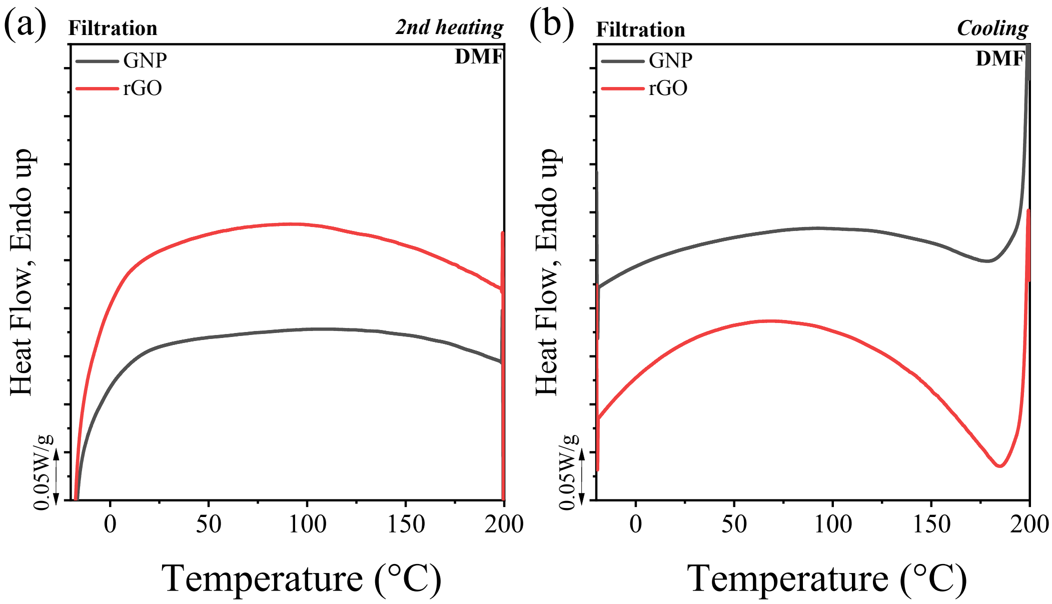

Figure S2. DSC curves of GNP and rGO nanopapers prepared via the filtration method. (a) 2nd heating, and (b) cooling. Observed plots are consistent with the instrumental baseline

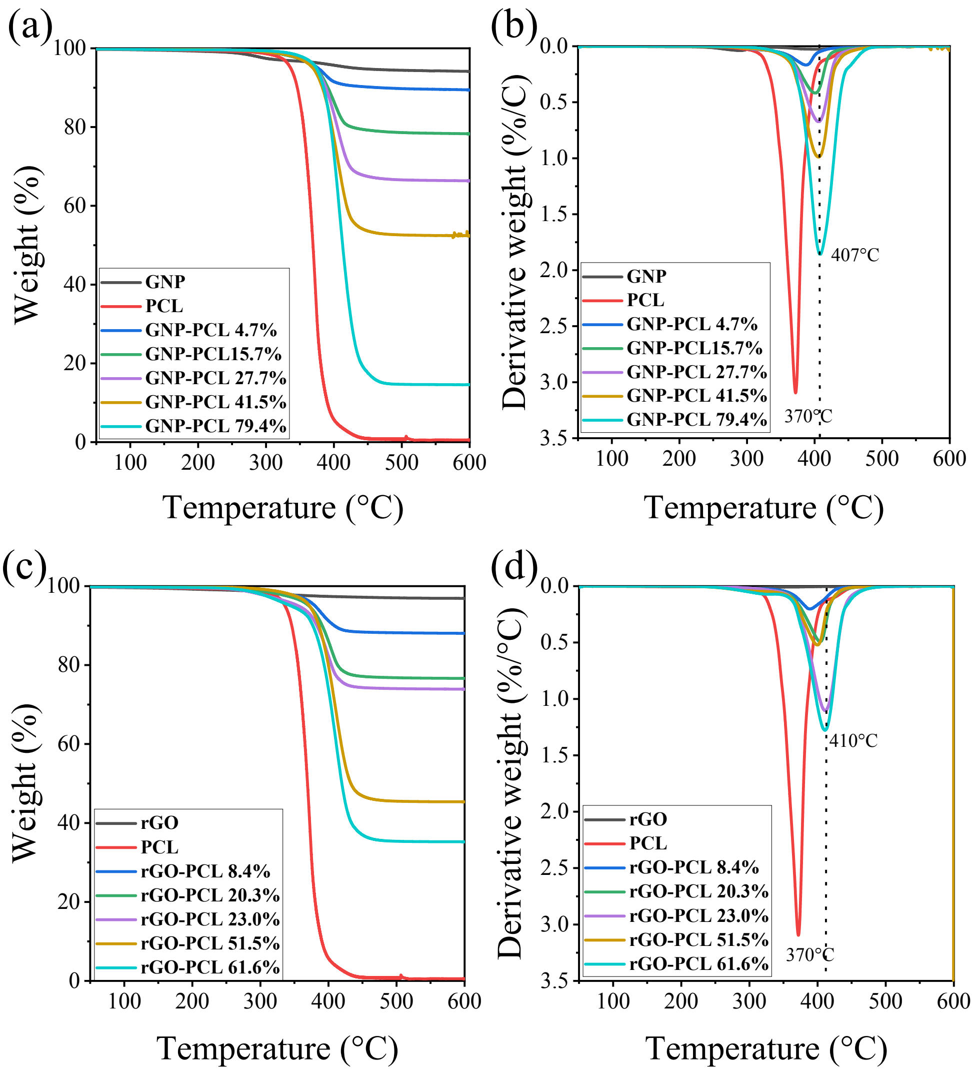

Figure S3. Thermogravimetric analysis curves of nanopaper prepared with GNP of different proportions of PCL, (a) weight (TG), (b) weight loss rate (DTG). Thermogravimetric analysis curves of nanopaper prepared with rGO of different proportions of PCL, (c) TG, (d) DTG. The samples for this test were derived from the DSC test samples.

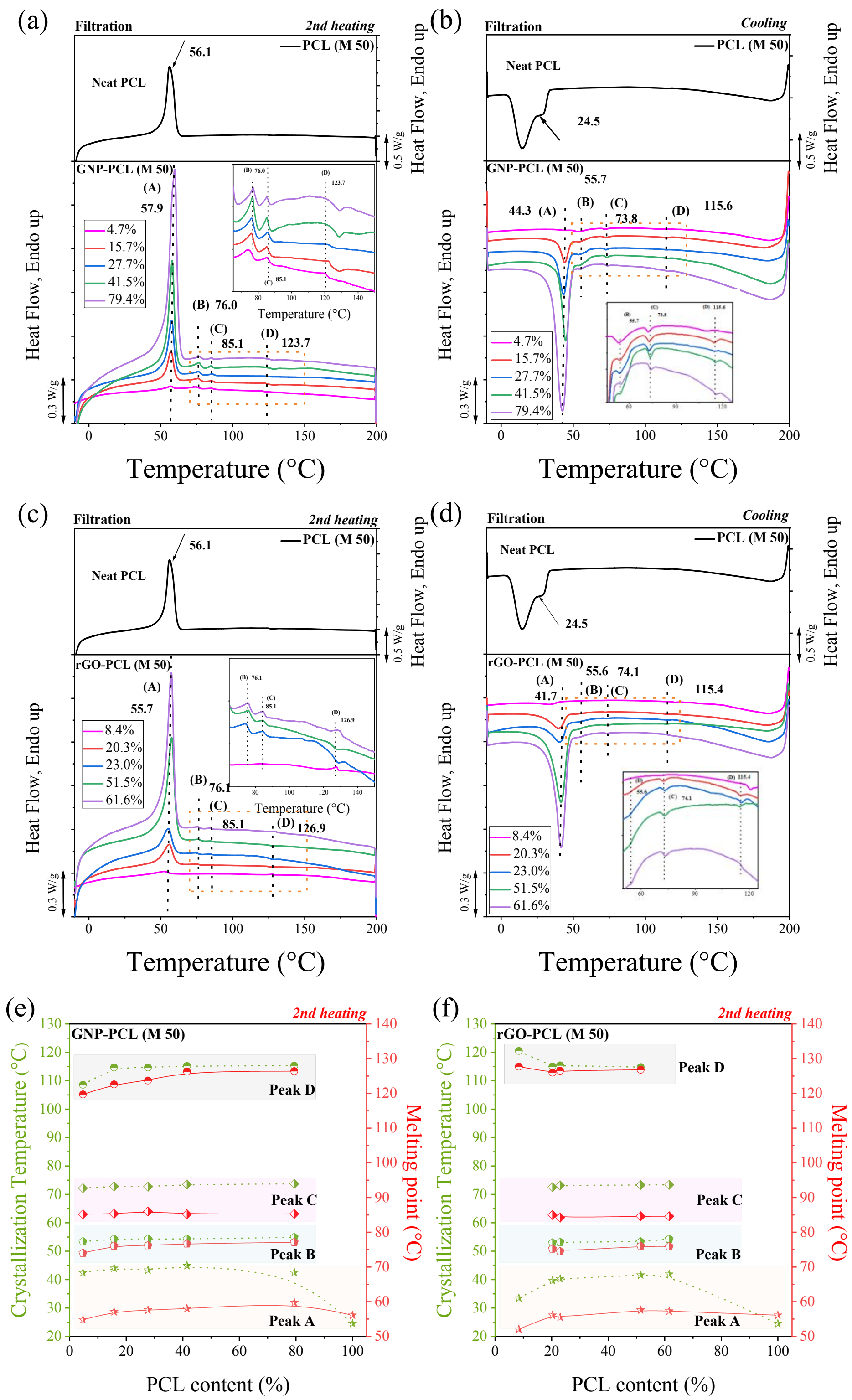

Figure S4. DSC curves (raw data) from the second heating and cooling curve with different PCL content for GNP (a), (b) and rGO (c), (d). Inset shows the magnification of the low intensity peaks. The relationship between the melting and cooling temperature of the different peaks with the content of PCL for nanopapers based on GNP (e) and rGO (f).

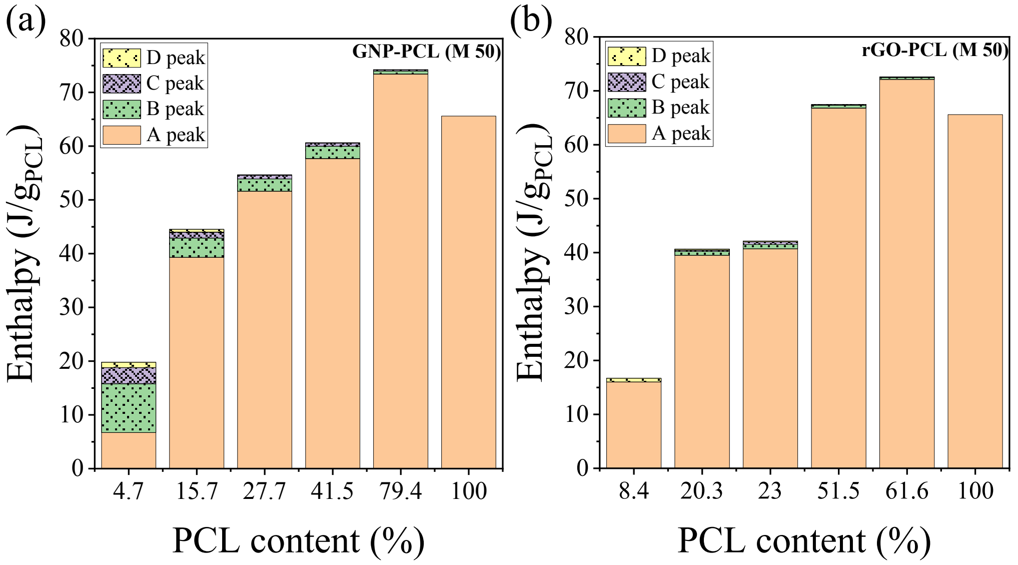

Figure S5. Integral enthalpy values of the endothermic peaks obtained during the 2nd DSC heating scans for the different nanopapers, as a function of the PCL content for (a) GNP, (b) rGO.

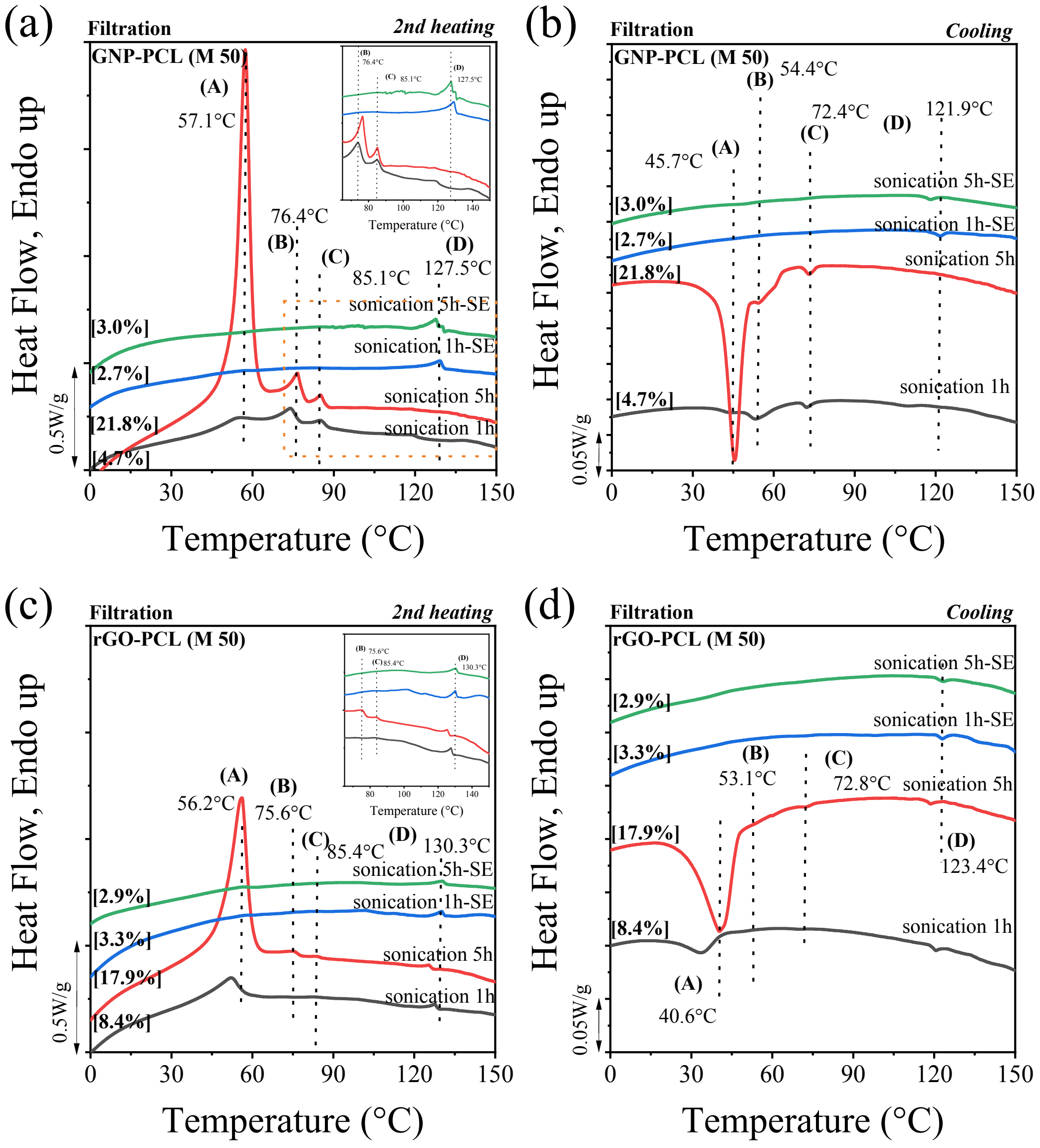

Figure S6. DSC curves from the second heating curve with selected nanopapers based on GNP (a) and rGO (b). In each graph, preparation conditions are reported on the right and PCL content is reported on the left. Inset shows the magnification of the low intensity peaks. SE identifies Soxhlet-extracted nanopapers

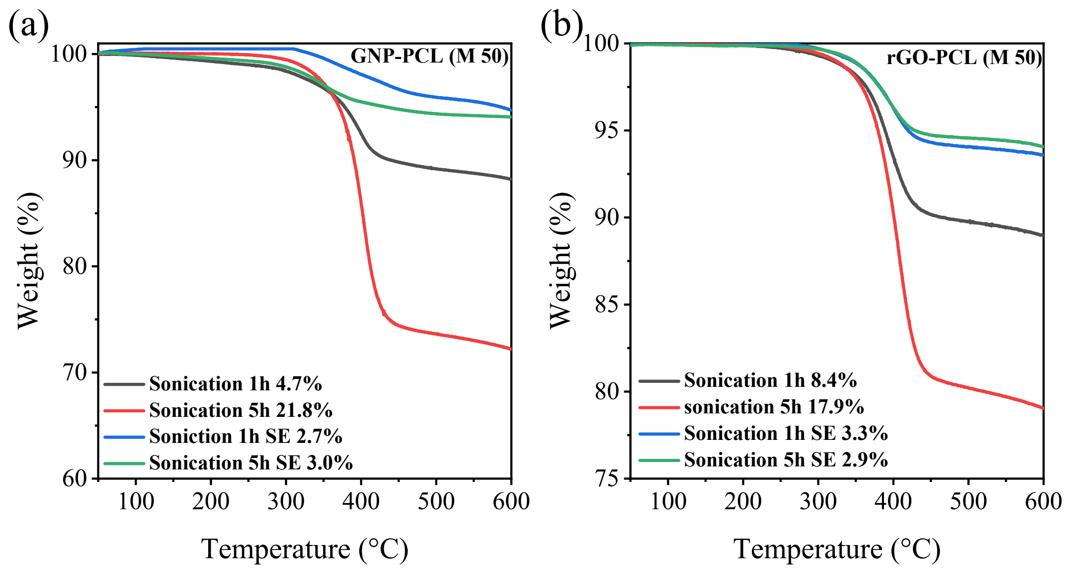

Figure S7. Thermogravimetric analysis curves (TG) of nanopapers prepared with different sonication time, based on (a) GNP, (b) rGO.

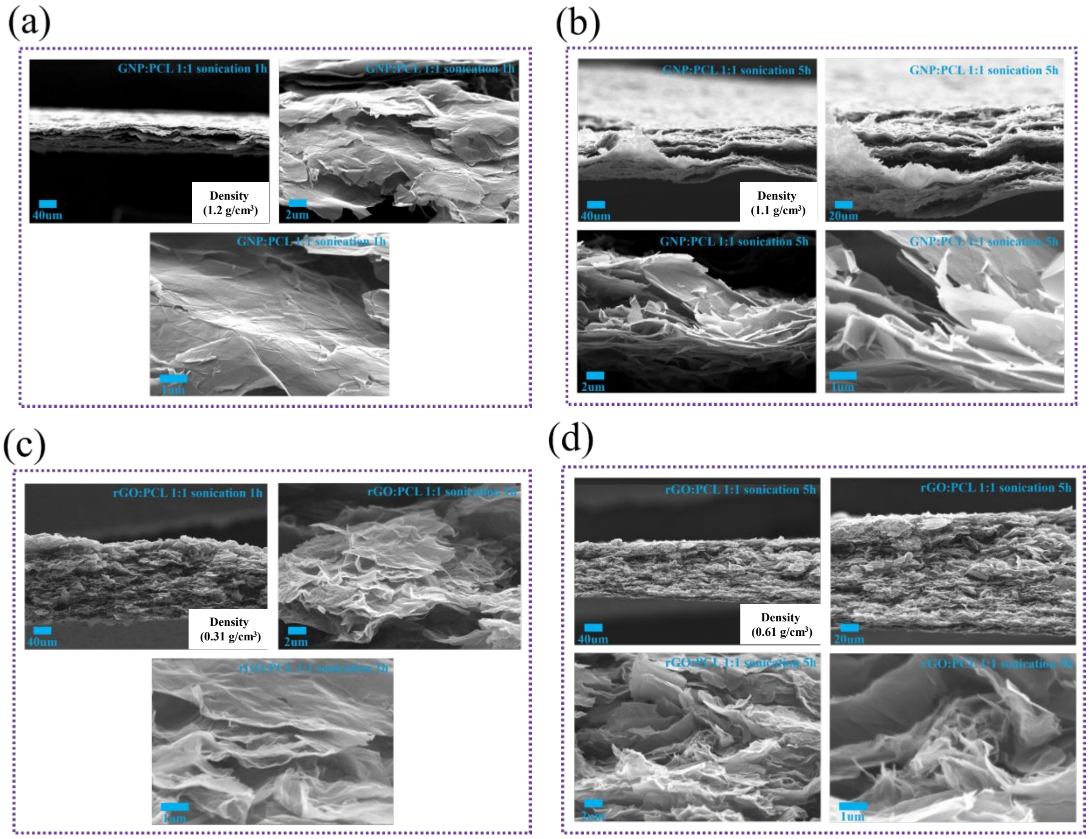

Figure S8. Cross-sectional SEM images of nanopapers with different sonication time (a) GNP (4.7%) 1h, (b) GNP (21.8%) 5h, (c) rGO (8.4%) 1h, (d) rGO (17.9%) 5h. The sample density is marked in the white box.

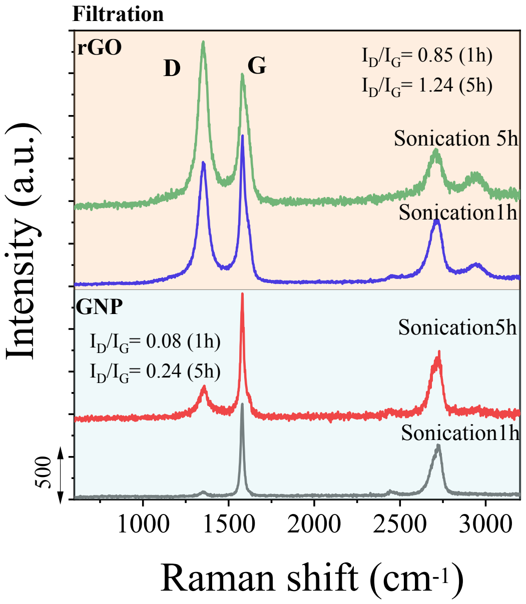

Figure S9. Raman spectra for nanopapers of GNP and rGO with different sonication time.

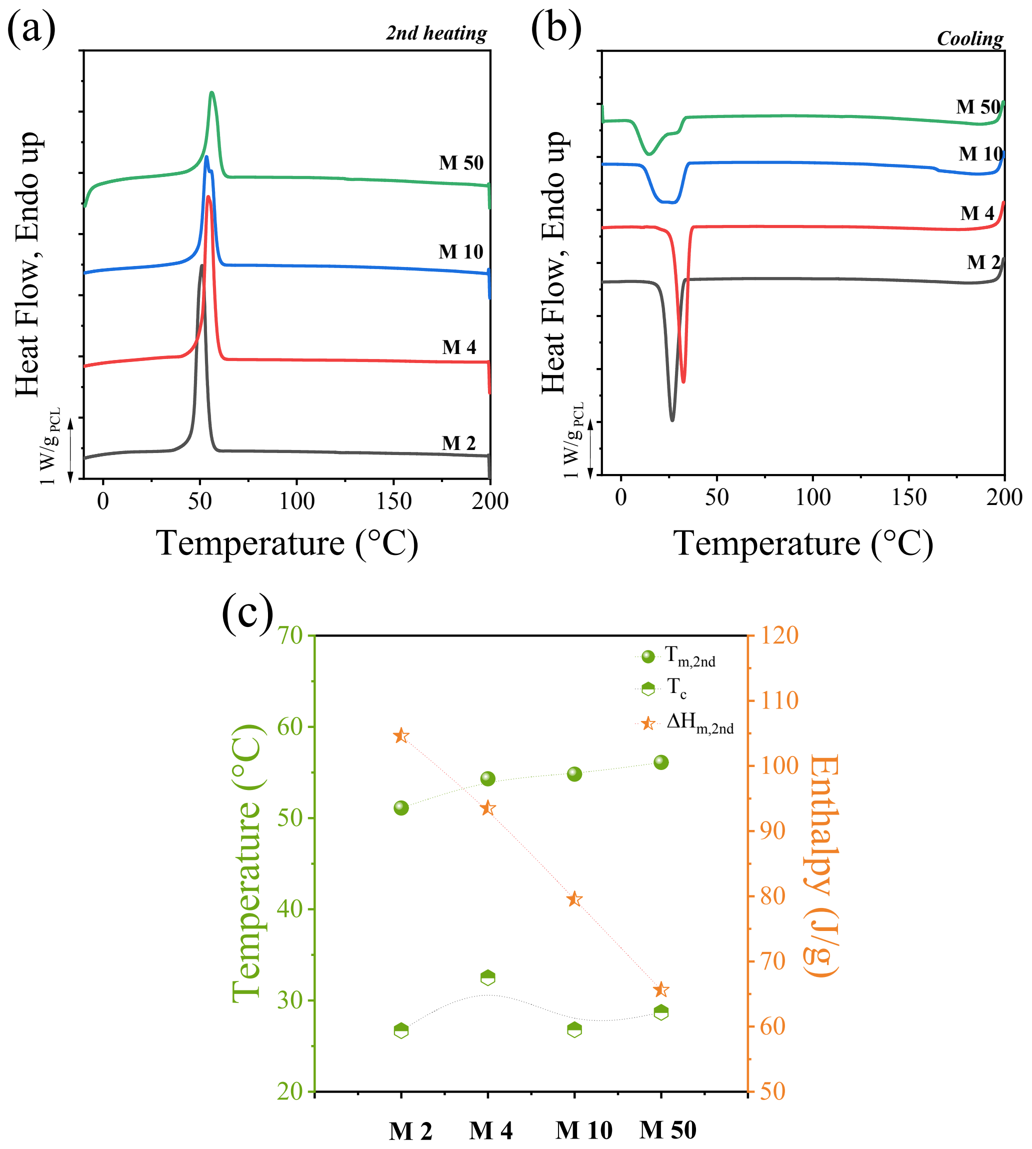

Figure S10. The DSC curves of different PCL with different molecular weight. (a) 2nd heating, (b) cooling. (c) The relationship of T_m, T_c and ΔH_m with different PCL molecular weight.

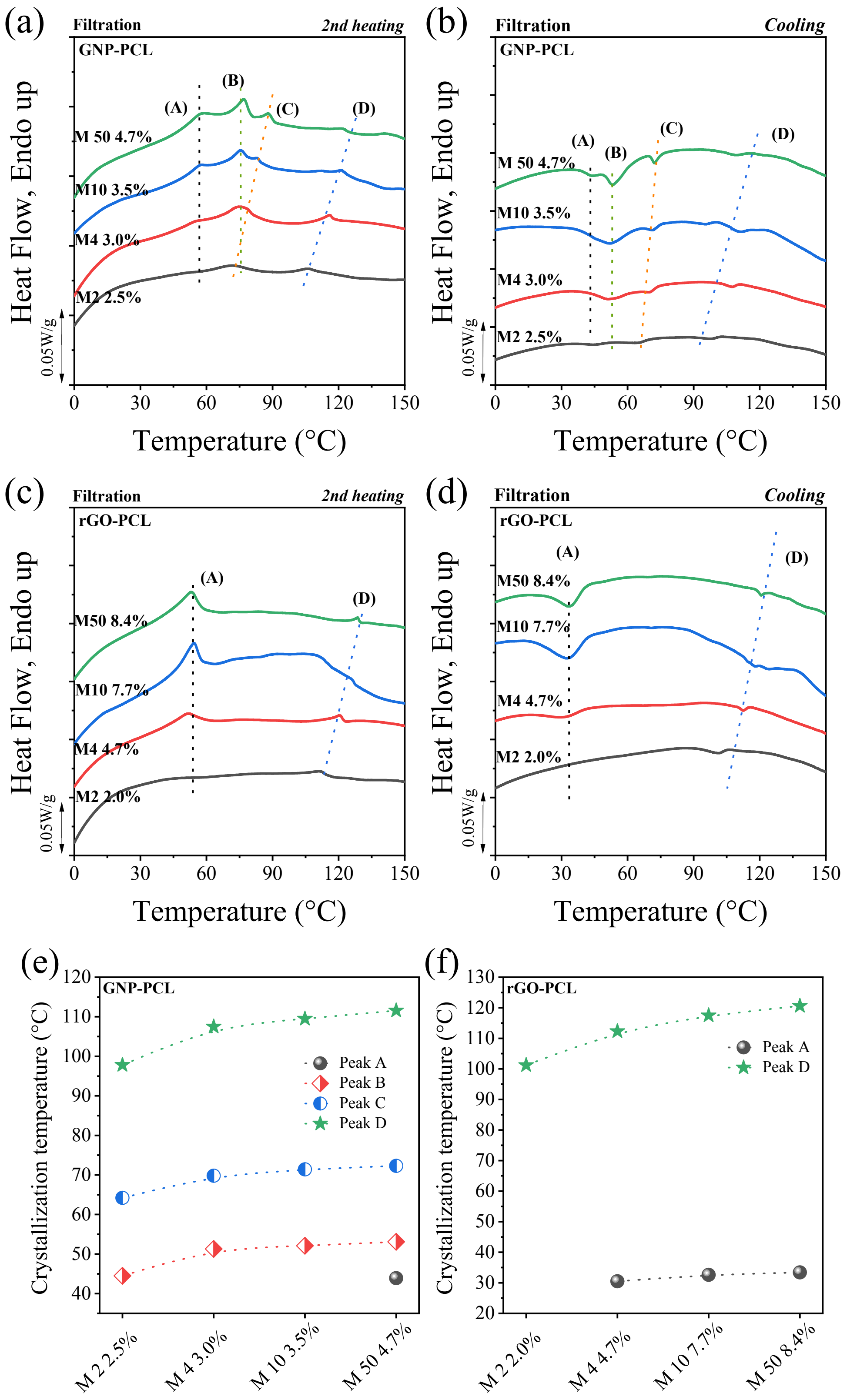

Figure S11. DSC curves (raw data) from the second heating and cooling curve with different PCL molecular weight for GNP (a), (b) and rGO (c), (d). The relationship between the melting and cooling temperatures of the different peaks with the molecular weight of PCL for nanopapers based on GNP (e) and rGO (f)

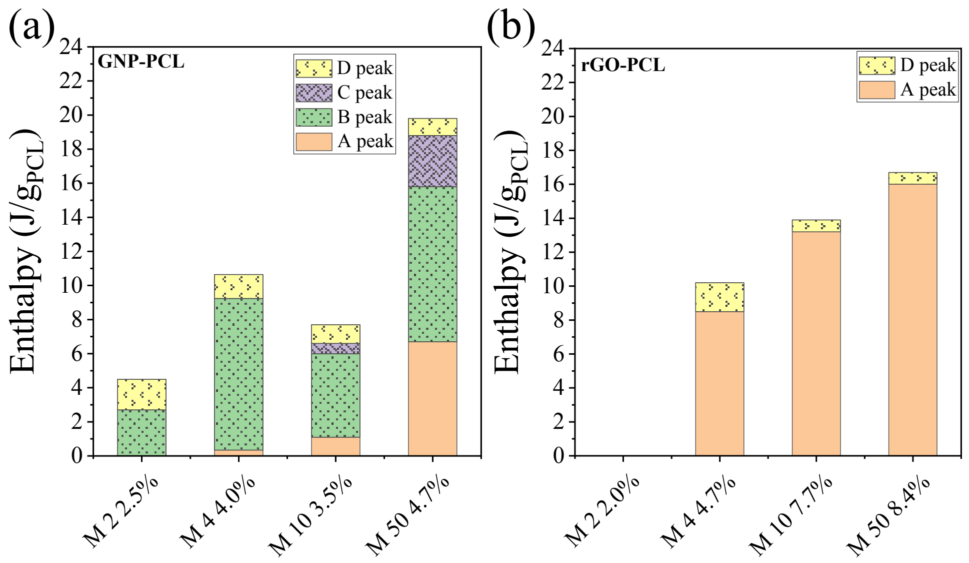

Figure S12. Integral enthalpy values of the endothermic peaks obtained during the 2nd DSC heating scans for the different nanopapers, as a function of the molecular weight for (a) GNP, (b) rGO.

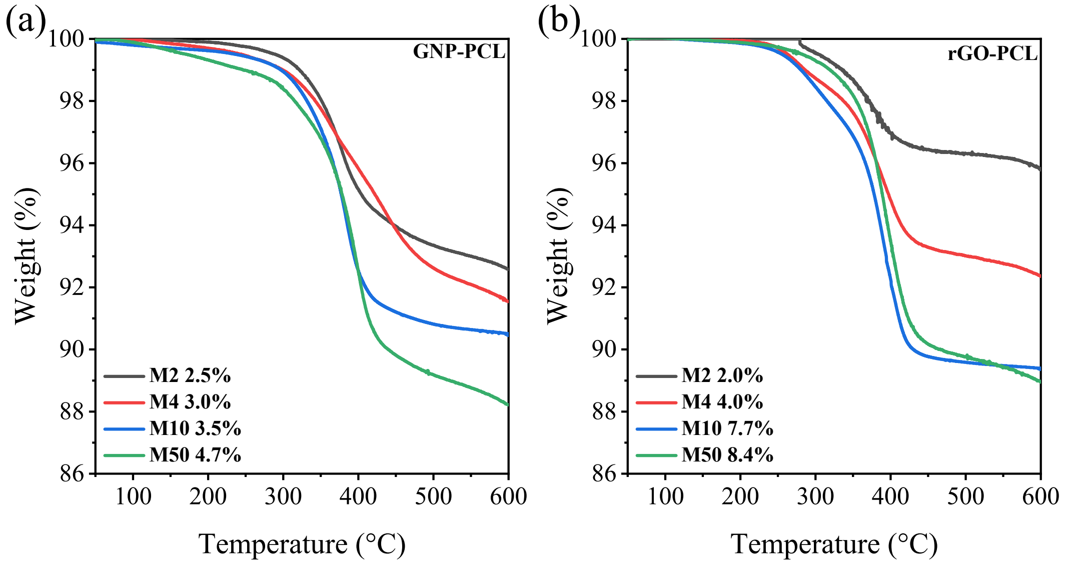

Figure S13. Thermogravimetric analysis curves of nanopapers with PCL of different molecular weight, (a) GNP, (b) rGO.

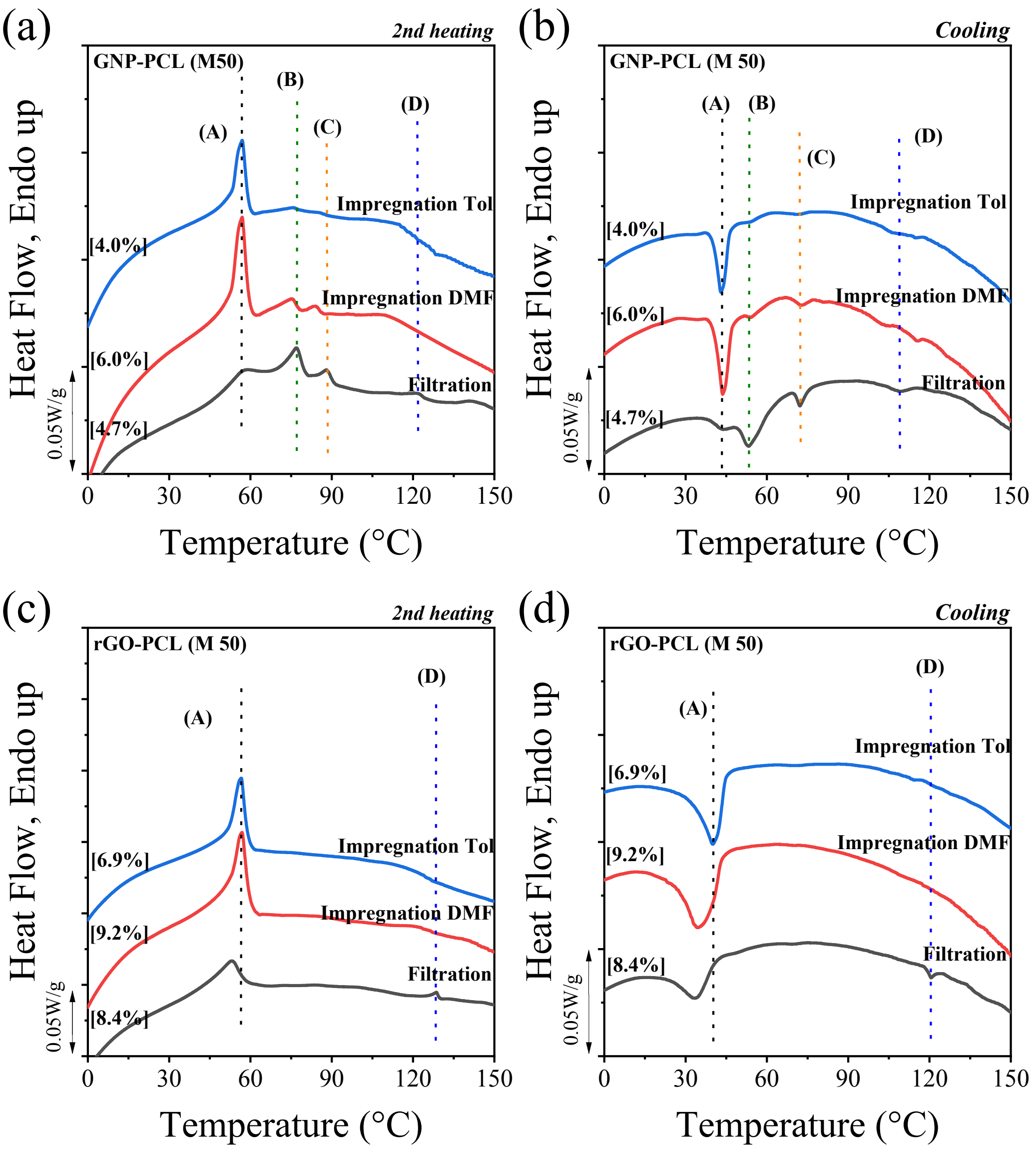

Figure S14. DSC curves (raw data) from the second heating and cooling curve with different preparation methods for GNP (a), (b) and rGO (c), (d).

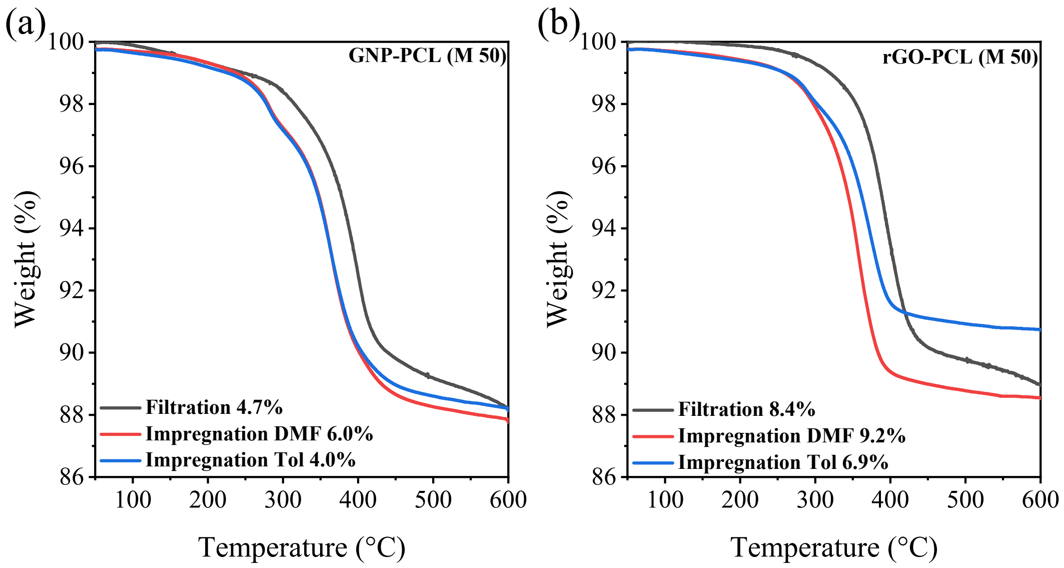

Figure S15. Thermogravimetric analysis curves of nanopapers obtained by different preparation methods, (a) GNP, (b) rGO.

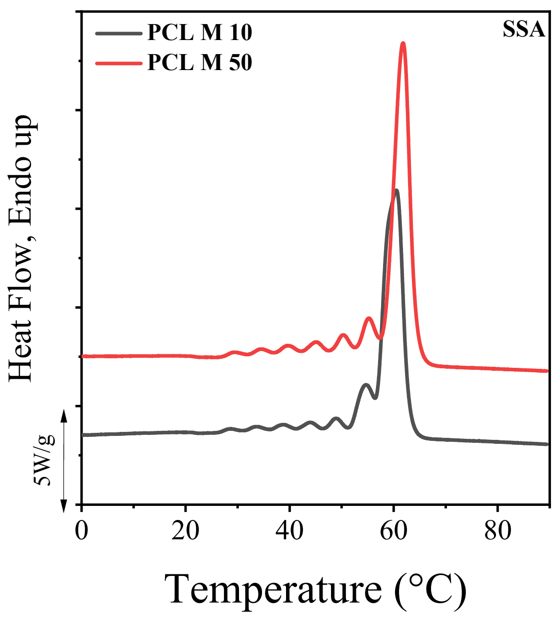

Figure S16. SSA final heating for the neat PCL with different molecular weight.

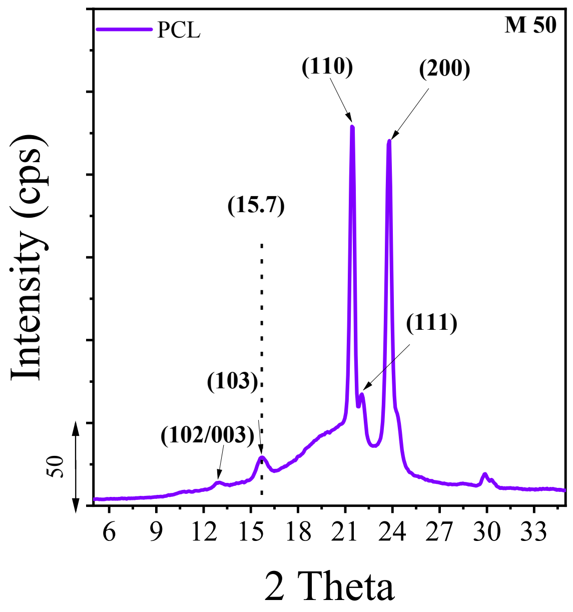

Figure S17. The X-ray diffraction patterns of PCL.

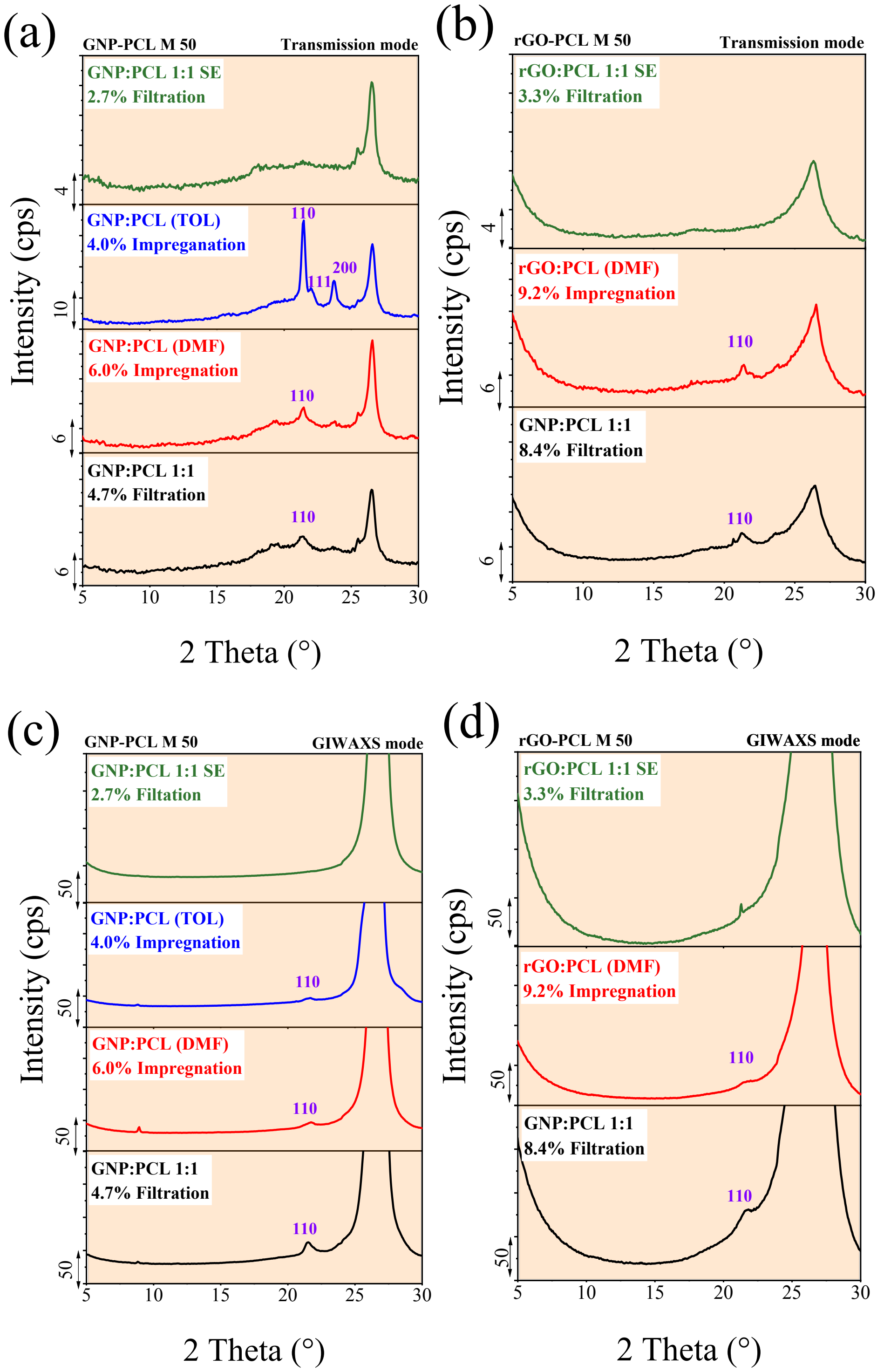

Figure S18. One-dimensional WAXS curves of a series of nanopapers with different test directions (a) GNP, and (b) rGO with in transmission mode, (c) GNP and (d) rGO with GIWAXS mode. The actual PCL content, reported in square brackets. SE is for Soxhlet-extracted nanopapers.

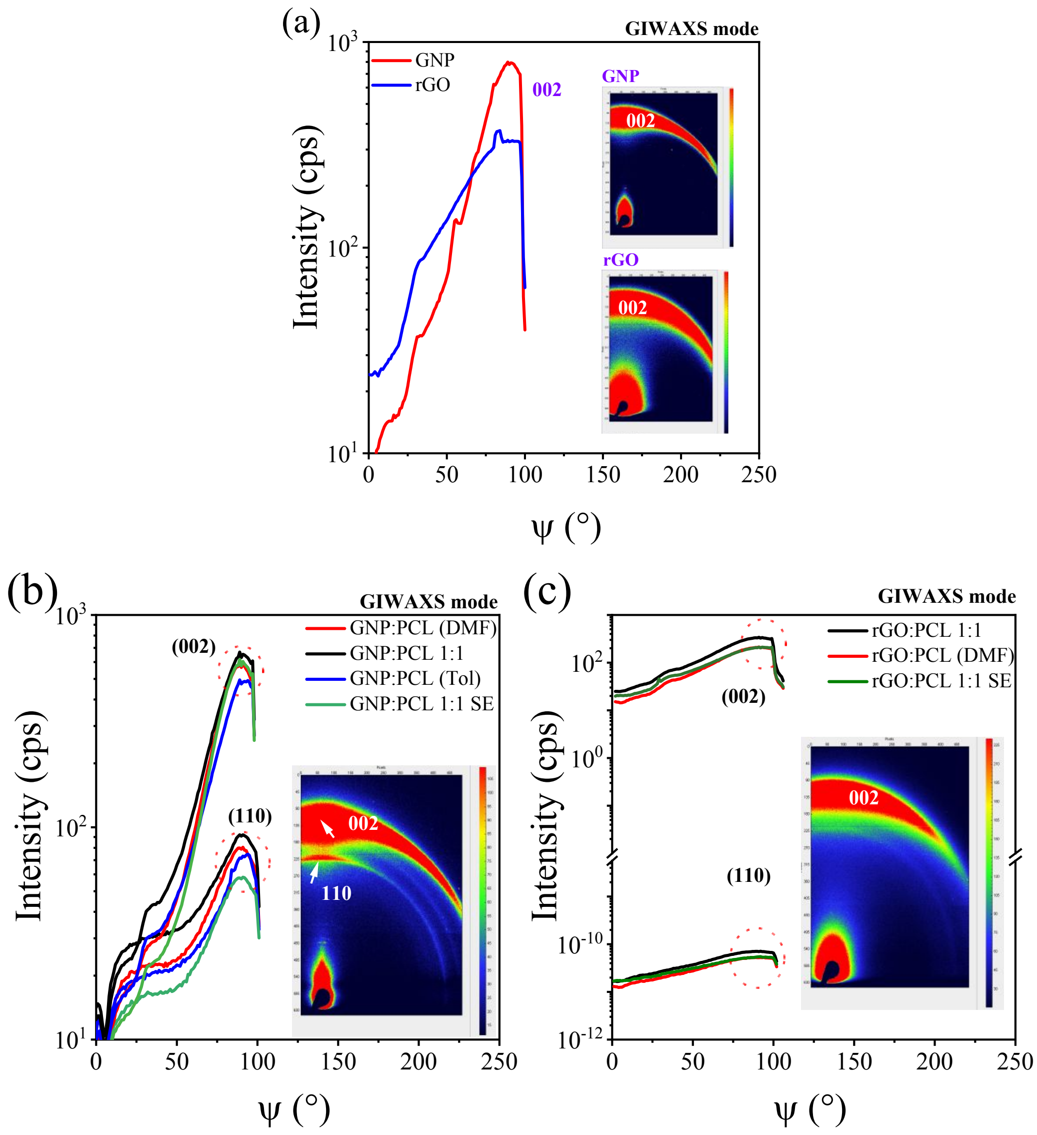

Figure S19. GIWAXS mode orientation intensity distribution of selected reflections vs. azimuthal angle for GNP and rGO nanopapers: a) (002) for pristine GRM nanaopapers, b) (110) for PCL vs (002) in GNP/PCL nanopapers and c) (110) for PCL vs (002) in rGO/PCL nanopapers.

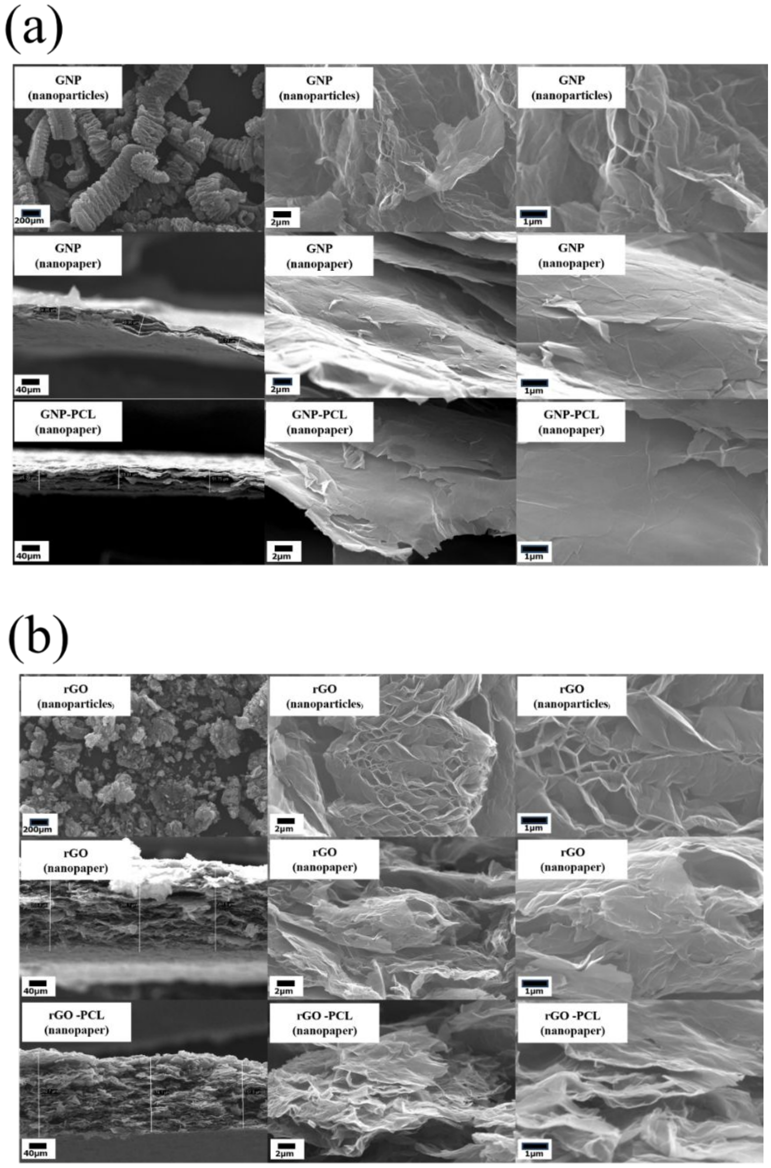

Figure S20. SEM pictures from GRM powders, pristine GRM nanopapers and GRM/PCL nanopapersbased on a) GNP and b) rGO.

To further investigate the change in porosity for nanopapers in the presence of PCL, nanopapers containing low PCL fractions[1] were measured for surface area by BET adsorption. Results showed a decrease in surface area from 19 to 15 m^2/g in GNP (Figure S1a) and from 70 to 46 m^2/g in rGO (Figure S1b). This reduction in the presence of PCL is attributed to a decrease in pore volume across the entire porosity range, as clearly evidenced by NLDFT pore size analysis, while Soxhlet extraction of PCL led to only a partial recovery of the initial porous volume (Figure S1c).

[1] Selected nanopapers are GNP:PCL 1:1 and rGO:PCL 1:1, both obtained by filtration with M50 PCL. See Table for characterization.

Table S3. The thermal conductivity of nanopapers obtained with different contents, molecular weights, and preparation methods

Sample	Molecular weight	PCL content (%)	Density (g/cm^3)	Thermal diffusivity (mm^2/s)	Thermal conductivity (W/m·K)
GNP		0	0.90±0.07	169.9±5.6	108.0±12.8
GNP/PCL (Filtration)	M50	4.7	1.20±0.10	148.6±1.9	137.4±13.4
		7.6	1.24±0.07	125.7±4.6	125.9±12.0
		11.6	1.26±0.09	130.9±7.3	141.8±18.6
		14.3	1.15±0.10	120.1±6.9	123.5±18.6
		39.0	1.25±0.14	91.6±6.0	138.9±25.7
	M10	3.5	1.35±0.08	158.1±2.0	161.2±11.7
	M4	4.0	1.15±0.05	152.7±8.1	133.7±13.3
	M2	2.5	1.15±0.11	157.2±4.4	134.2±15.9
GNP/PCL (Impregnation DMF)	M50	5.0	1.12±0.03	143.7±7.8	124.7±10.2
rGO		0	0.32±0.02	40.5±1.7	8.4±1.0
rGO/PCL (Filtration)	M50	8.4	0.31±0.03	39.7±3.4	10.1±1.9
		10.7	0.41±0.02	30.2±1.7	10.5±0.5
		17.9	0.39±0.05	31.6±1.0	11.6±1.9
		18.2	0.43±0.05	28.7±1.1	11.7±1.8
	M10	8.5	0.34±0.01	37.0±1.4	9.4±0.7
	M4	4.7	0.31±0.03	40.5±1.5	9.7±0.3
	M2	2.0	0.34±0.01	37.6±0.6	9.4±0.4
rGO/PCL (Impregnation DMF)	M50	5.7	0.30±0.07	30.4±0.6	7.1±1.9

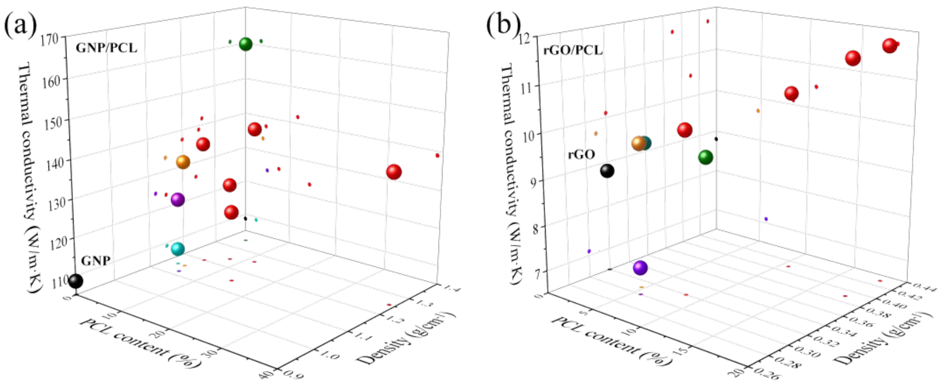

Figure S21. The relationship between thermal conductivity, density and content of PCL with different loadings, different molecular weights, and different preparation methods of nanopapers GNP (a) and rGO (b). Red, green, orange, and blue dots represent nanopaper with molecular weights of M50, M10, M4, and M2, respectively. The purple color represents nanopaper with a molecular weight of M50 prepared using the impregnation method, whereas in black are pristine GNP and rGO nanopapers.
